# Harnessing spiropyran isomerization in lanthanide metallopolymers for sequential logic encryption and anticounterfeiting

**DOI:** 10.1039/d5sc06759f

**Published:** 2025-10-27

**Authors:** Weixu Feng, Xiaolin Liao, Sumin Lu, Dong Han, Qianrong Guo, Yan Zhao, Wei Tian, Hongxia Yan

**Affiliations:** a Xi'an Key Laboratory of Hybrid Luminescent Materials and Photonic Device, School of Chemistry and Chemical Engineering, Northwestern Polytechnical University Xi'an 710129 Shaanxi China hongxiayan@nwpu.edu.cn

## Abstract

Stimuli-responsive lanthanide-based luminescent materials, exhibiting dynamic changes in photoluminescence in response to external stimuli and reversible recovery of their properties, hold great promise for advanced optical encryption and anticounterfeiting technologies. Nevertheless, simultaneously achieving reversible responsiveness to light, pH, and temperature alongside tunable multicolor emission remains a significant challenge. Herein, we present a facile strategy for constructing two terbium-based metallopolymers (Poly-Tb(1–2)) that exhibit triple-mode reversible responsiveness and tunable emission behavior. By copolymerizing spiropyran and terpyridine into a polymethyl methacrylate backbone and coordinating the polymer with β-diketone Tb(iii) complexes, Poly-Tb(1–2) with effective energy transfer from the Tb^3+^ ions to the merocyanine isomer of spiropyran were obtained. The resulting Poly-Tb(1–2) exhibit excellent photochromic behavior and pH-dependent emission color switching. Remarkably, Poly-Tb(1) exhibits reversible responsiveness to both light and pH stimuli, and in its MC state, shows the rare phenomenon of enhanced Tb^3+^ luminescence upon heating. Such multifunctional optical behavior enables precise multicolor modulation and exceptional adaptability to complex external stimuli. Capitalizing on their robust multi-stimuli responsiveness, these materials were further applied in erasable photopatterning and sequential logic information encryption systems. This work offers a new design strategy for lanthanide metallopolymers and may contribute to the development of next-generation intelligent multifunctional materials.

## Abstract 2

A copolymerization–coordination strategy yields Tb-based metallopolymers with reversible multi-stimuli responses, tunable multicolor emission, and rare negative thermal quenching, enabling erasable photopatterning and sequential logic encryption.

## Graphical abstract

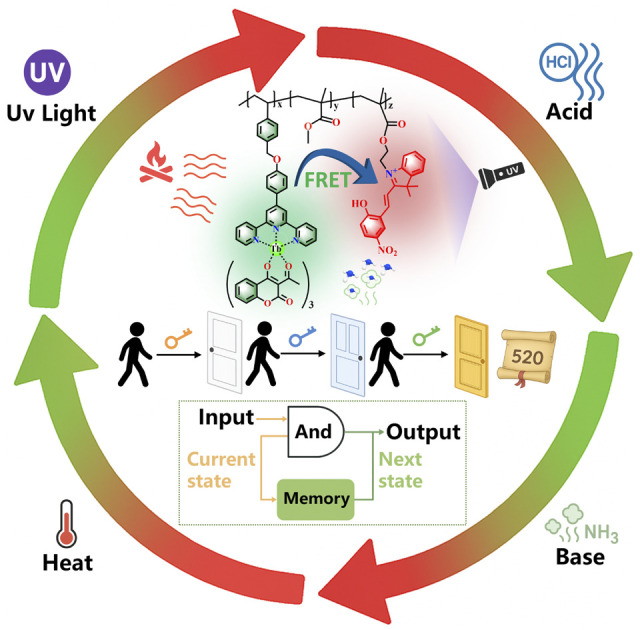

## 1 Introduction

In response to the escalating global demand for data security and information authenticity, stimuli-responsive luminescent materials with dynamic and reversible photoluminescence under external stimuli have attracted growing attention.^1,2^ Unlike conventional static fluorophores, these advanced systems can be precisely tailored to reversibly respond to a wide range of environmental cues, including light,^3–5^ heat,^6,7^ pH,^8,9^ and mechanical force.^10,11^ Such versatile responsiveness enables the seamless integration of luminescence with stimulus-induced chromic transitions, thereby enhancing the capacity for information encoding while endowing these materials with programmability and rewritability.^12–14^ Building upon these features, sequentially responsive systems whose outputs are governed not only by the type of stimuli but also by their temporal order have emerged as a compelling platform for logic-controlled data security.^15^ However, to achieve sequentially responsive functionality at the molecular level for practical encryption applications, stimuli-responsive luminescent materials that simultaneously exhibit reversible multi-stimuli responsiveness, tunable multicolor emission, robust structural stability, and excellent processability are required, which is still a considerable challenge.

Traditionally, achieving multicolor emission in responsive functional materials has primarily involved incorporating stimuli-sensitive luminophores through physical doping or covalent grafting methods. Representative luminophores include organic dyes,^16–18^ carbon quantum dots,^19,20^ and lanthanide (Ln^3+^) complexes.^21,22^ Among these, lanthanide complexes are particularly attractive due to their sharp emission bands, large Stokes shifts, high photoluminescence quantum yields, and prolonged excited-state lifetimes, making them ideal candidates for advanced photonic applications.^23,24^ To impart multi-environmental responsiveness to lanthanide ions, a widely adopted strategy involves coupling them with photoactive molecular switches such as diarylethenes (DAEs),^25–27^ spiropyrans (SPs),^28–30^ and azobenzenes (AZOs).^31,32^ These molecular switches dynamically modulate luminescence through Förster resonance energy transfer (FRET), thereby enabling precise external control over emission intensity and color. Among these, spiropyrans have attracted considerable interest owing to their well-defined reversible isomerization behavior and sensitivity to multiple external stimuli.^33–35^ Upon ultraviolet irradiation, SP undergoes a transition from a closed-ring, colorless, non-emissive form to an open-ring merocyanine (MC) isomer with extended π-conjugation, vivid coloration, and enhanced fluorescence. Furthermore, this isomerization is highly sensitive to variations in pH and temperature, thus underscoring the exceptional potential of SP-based lanthanide systems for sequential logic encryption applications. However, despite significant advancements in developing stimuli-responsive materials based on lanthanide complexes and spiropyran units, several critical challenges hinder their practical applications. On the one hand, conventional lanthanide complexes typically exist as powders or crystals, significantly limiting their processability and integration into functional device architectures. On the other hand, strong intermolecular packing of SP molecules in the solid state reduces their photochromic responsiveness, thereby diminishing their sensitivity to external stimuli. To overcome these challenges, lanthanide-containing metallopolymers incorporating SP units have emerged as promising hybrid materials.^36–39^ These polymeric systems offer superior solution processability, structural tunability, mechanical durability, and enhanced functional versatility. Crucially, their polymeric frameworks provide a flexible platform that allows spatial organization and effective modulation of luminescent moieties, thus optimizing photophysical properties under complex stimulus conditions. Nevertheless, lanthanide luminescence remains inherently susceptible to environmental perturbations such as acidic or thermal stress, which can disrupt energy transfer pathways and substantially quench emission intensity.^40–42^ Consequently, the rational design of lanthanide-containing metallopolymers exhibiting robust reversible fluorescence chromic responses to multiple external stimuli, including light, heat, and pH, constitutes an underexplored yet highly attractive research frontier.

**Fig. 1:**
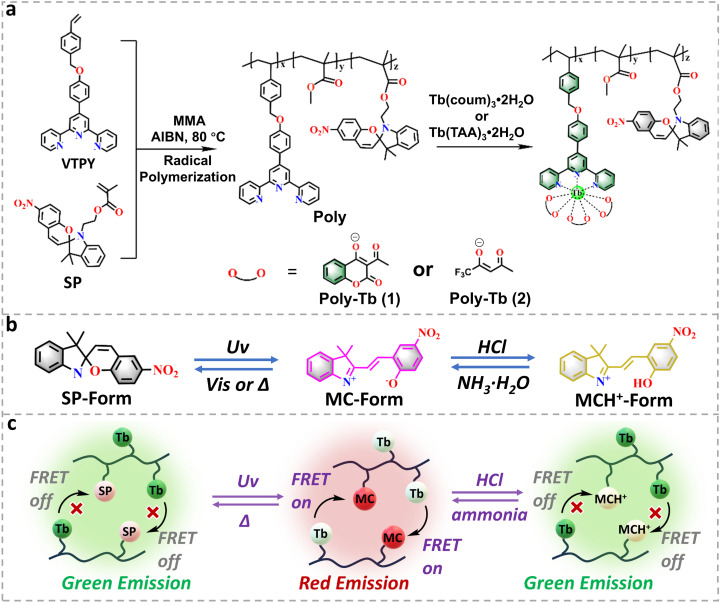
(a) Synthetic procedures for Poly and Poly-Tb(1–2); (b) schematic illustration of the spiropyran moiety's response mechanisms to light, temperature, and acid/base stimuli; (c) multi-stimuli-responsive behavior of the lanthanide metallopolymer system.

In this work, we present the design and synthesis of a novel class of terbium-based luminescent metallopolymers exhibiting triple-mode reversible responsiveness to light, pH, and temperature. By copolymerizing spiropyran and terpyridine monomers into a polymethyl methacrylate backbone, terpyridine ligands are strategically introduced as effective coordination sites for Tb^3+^ ions. Subsequent functionalization with two distinct β-diketone Tb(iii) complexes results in two well-defined metallopolymer systems with tunable optical properties (Fig. 1a). First, the spiropyran units exhibit reversible responses to external stimuli, including light, temperature, and pH (Fig. 1b). Second, the substantial spectral overlap between the MC absorption of spiropyran and the Tb^3+^ emission facilitates efficient FRET. This dual effect enables the material to achieve reversible emission color switching under varying light, pH, and temperature conditions (Fig. 1c), significantly expanding its functional versatility. Importantly, by judiciously tuning the energy levels of β-diketone ligands, the thermal response of the systems is finely modulated. Notably, Poly-Tb(1) in the MC state exhibits a negative thermal quenching effect, in which the luminescence intensity of Tb^3+^ increases upon heating, which is seldom observed in lanthanide complex systems. This unique behavior facilitates reversible thermochromism, adding a novel dimension to the materials' adaptive responses. Exploiting these multifunctional optical characteristics, we further illustrate their potential applications in high-security platforms, including erasable optical writing films and programmable sequential logic encryption systems incorporating self-erasing and anti-brute-force functionalities.

## 2 Results and discussion

### 2.1. Rational design and characterization of multi-stimuli-responsive Tb^3+^-metallopolymers

Our approach to developing multi-stimuli-responsive lanthanide metallopolymers relies on the strategic integration of photochromic SP units and luminescent terbium complexes within a poly(methyl methacrylate) (PMMA) polymer matrix (Fig. 1a). In this carefully constructed architecture, SP moieties are covalently incorporated into the polymer backbone to serve as responsive switches under photo stimulation. Concurrently, terpyridine ligands (VTPY) are designed to effectively chelate Tb^3+^ ions, thereby conferring the metallopolymer with the characteristic green emission from Tb^3+^ ions. To further enhance luminescent efficiency, β-diketonate ligands were specifically employed to finely tune the energy alignment between the organic ligand triplet states and the ^5^D_4_ excited state of Tb^3+^ ions. This deliberate design significantly amplifies the antenna effect and facilitates efficient FRET from the Tb^3+^ centers to spiropyran units. The intrinsic amorphous structure of the PMMA matrix provides substantial conformational freedom essential for reversible spiropyran-to-merocyanine (SP–MC) photoisomerization, contributing critically to the polymer's pronounced stimuli-responsiveness in the solid state.^36^

**Fig. 2:**
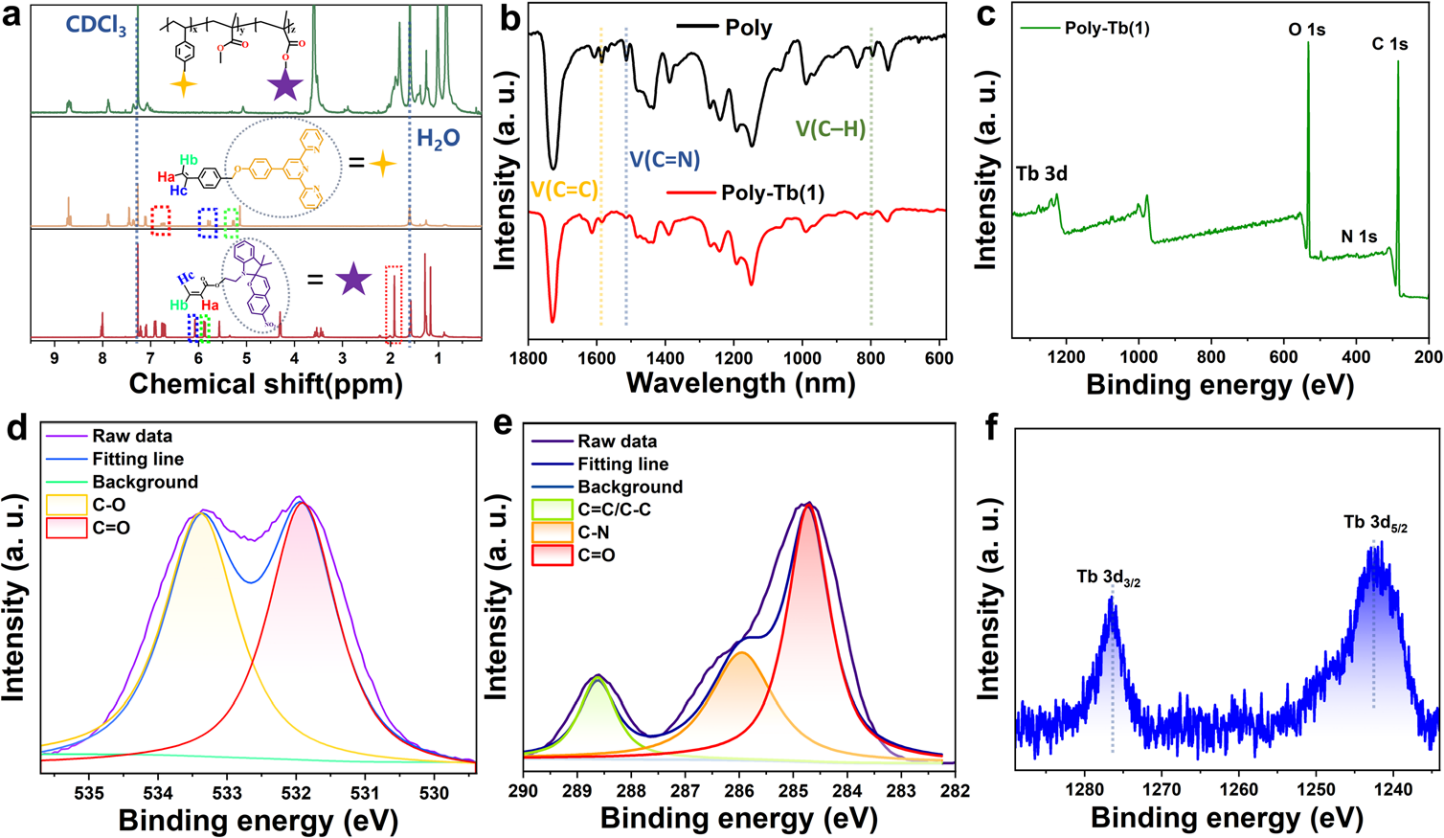
Structural characterization of Poly and Poly-Tb(1): (a) comparison of ^1^H NMR spectra of VTPY, SP and Poly; (b) FTIR spectra of Poly and Poly-Tb(1); (c) XPS survey spectrum of Poly-Tb(1); (d–f) high-resolution XPS spectra of C 1s, O 1s, and Tb 3d regions, respectively.

The synthetic pathways and schematic depictions of the non-coordinated polymer (Poly) and its terbium-coordinated derivatives (Poly-Tb(1–2)) are illustrated in Fig. 1a. SP and VTPY were synthesized according to previously published protocols,^43,44^ with comprehensive details outlined in the SI. The parent polymer backbone (Poly) was synthesized through free-radical copolymerization of VTPY, SP, and MMA with a feed molar ratio of 1 : 4 : 100. Subsequent coordination reactions of Poly with Tb(Coum)_3_·2H_2_O (Coum: 3-acetyl-4-hydroxy-coumarin) and Tb(TAA)_3_·2H_2_O (TAA: (*Z*)-1,1,1-trifluoro-4-oxopent-2-en-2-olate) yielded the luminescent and photochromic metallopolymers Poly-Tb(1) and Poly-Tb(2), respectively. Additionally, the control polymers (Poly-Tb(R1–2)) lacking the SP component were also synthesized to investigate the mechanism of the energy transfer dynamics and multi-stimuli responsiveness (Scheme S1). Detailed synthetic procedures and structural characterization data are provided in the SI. Extensive structural characterization of the monomers and Poly-Tb(1–2) was performed using an array of analytical techniques, including ^1^H NMR, FT-IR, GPC, PXRD, XPS, and TGA. As shown in Fig. S1–S5, the ^1^H NMR spectra confirmed the successful synthesis and expected chemical structures of the synthesized SP, VTPY and Coum. Following polymerization, the disappearance of vinyl proton signals in the ^1^H NMR spectrum of Poly compared to VTPY and SP (Fig. 2a), confirmed the complete polymerization of the monomers, validating the efficiency of the free-radical copolymerization process. Quantitative analysis of characteristic proton resonances corresponding to VTPY, SP and MMA indicated an actual polymer composition of approximately 1 : 3 : 70. The deviation from the initial feed ratio is likely due to steric hindrance introduced by bulky aromatic substituents during polymerization.^45^ FT-IR spectroscopy provided additional evidence supporting the structural integrity and metal coordination within the polymer system (Fig. 2b and S6). A prominent absorption at 1730 cm^−1^ corresponding to ester C=O stretching vibrations was consistently observed across Poly and Poly-Tb(1–2). Notably, the characteristic out-of-plane C–H bending vibration of pyridine rings at 796 cm^−1^ diminished significantly upon coordination, confirming successful chelation of Tb^3+^ ions with terpyridine nitrogen atoms.^46^ Further attenuation in aromatic skeletal vibrations at 1584 and 1515 cm^−1^ substantiated ligand-to-metal coordination accompanied by structural reorganization within the polymer backbone.

Subsequently, the PXRD patterns of Poly and Poly-Tb(1-2) (Fig. S7) displayed broad halos devoid of discrete diffraction peaks, confirming their amorphous nature and suggesting that no detectable crystalline Tb(Coum)_3_·2H_2_O or Tb(TAA)_3_·2H_2_O domains are present. GPC analysis showed molecular mass distributions with dispersities of 1.80 for Poly-Tb(1) and 2.10 for Poly-Tb(2), along with number-average molecular mass (Mn) of 12.70 kDa and 13.20 kDa, respectively (Fig. S8 and Table S1). XPS analysis further confirmed the polymer composition, showing characteristic signals for C 1s (284 eV), N 1s (399 eV), and O 1s (531 eV) (Fig. 2c–f, S9 and 10). Crucially, distinct signals at 1246 eV and 1272 eV corresponded to the Tb^3+^ 3d_5/2_ and 3d_3/2_ spin–orbit components. Moreover, elemental atomic percentages calculated from the peak integration areas revealed that the ratio of Tb to N is close to 1 : 6, which is consistent with the intended compositional stoichiometry of the polymer. These results provide further evidence for the successful incorporation of Tb^3+^ into the polymer backbone. Thermal stability assessed *via* TGA (Fig. S11) indicated robust performance, with a two-step mass-loss profile observed for Poly and Poly-Tb(1–2): an initial minor weight loss below 261 °C, attributed to residual solvents or moisture, followed by a significant decomposition phase between 261 °C and 450 °C, indicative of polymer backbone degradation. These results confirm the successful synthesis of the metallopolymer and highlight its excellent thermal stability, a critical attribute for practical applications requiring reliable stimuli-responsive performance.

### 2.2. Photoresponsive characteristics of Tb^3+^-metallopolymers

**Fig. 3:**
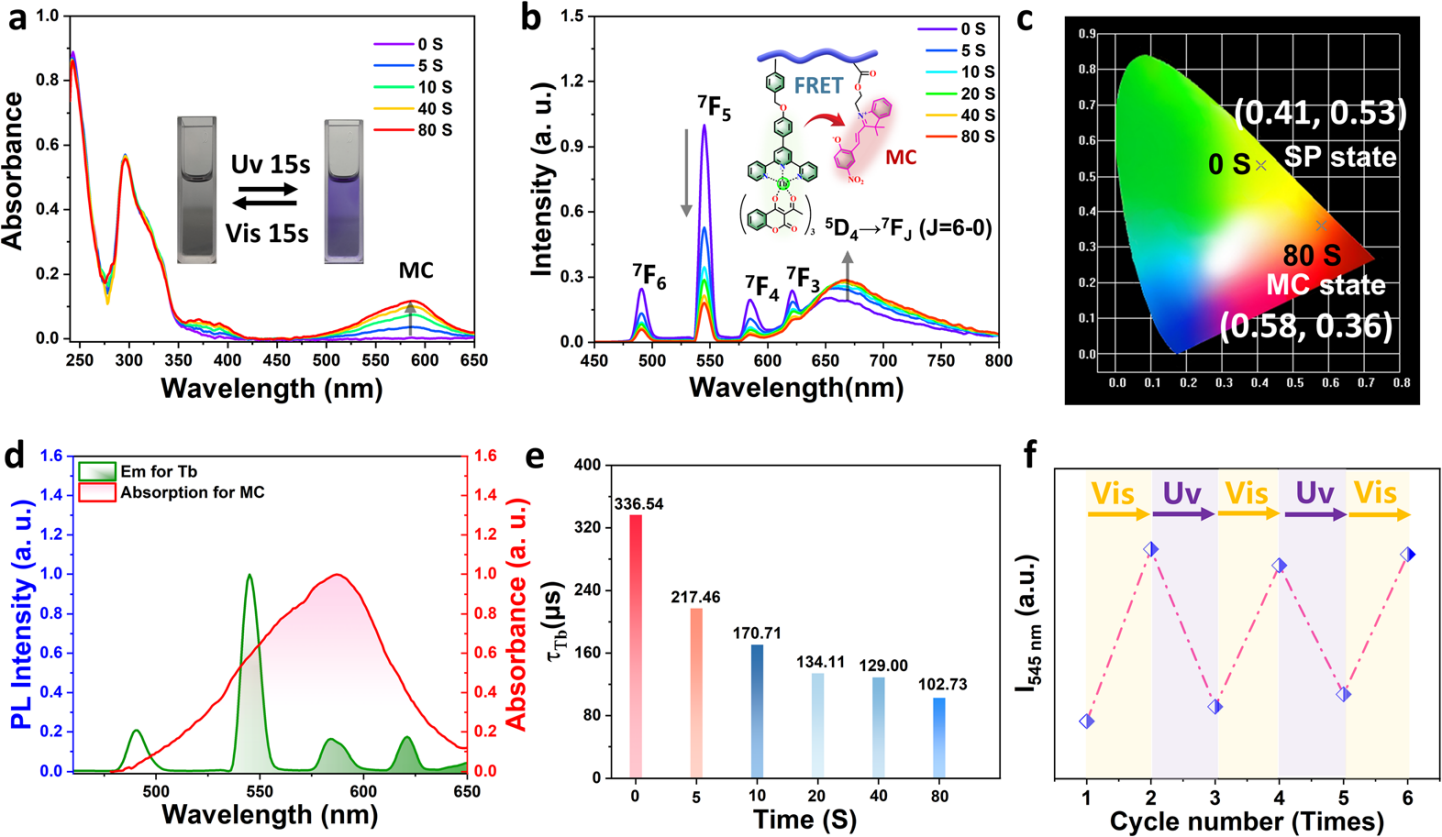
Photo-responsive behavior of Poly-Tb(1): (a) UV-vis absorption spectra of Poly-Tb(1) in dichloromethane under different durations of UV irradiation; (b) photoluminescence spectra of solid-state Poly-Tb(1) under 365 nm UV light irradiation (excitation at 310 nm); (c) corresponding CIE chromaticity coordinates; (d) comparison between the emission spectrum of Tb^3+^ and the absorption spectrum of the MC form; (e) luminescence lifetime changes of Poly-Tb(1); (f) fluorescence intensity at 545 nm of Poly-Tb(1) during cyclic exposure to UV/vis radiation.

To elucidate the dynamic photoluminescence behavior of the Tb^3+^-based metallopolymers, we carried out a comprehensive investigation into the photophysical properties of Poly-Tb(1) and Poly-Tb(2). The UV-vis absorption spectra of these metallopolymers in dichloromethane (2 mg mL^−1^) revealed distinct absorption bands centered at approximately 291 nm and 296 nm (Fig. 3a and S12), which are attributed to the π–π* transitions of aromatic chromophores embedded within the polymer backbone.^47^ Upon exposure to 365 nm ultraviolet irradiation, the SP units embedded in the polymer matrix underwent efficient photoisomerization to their open-ring MC form. This photochemical transformation was evidenced by the gradual appearance and intensification of two new absorption bands at 380 nm and 588 nm in the time-dependent UV-vis spectra. These bands are assigned to π–π* transitions and intramolecular charge transfer (ICT) processes associated with the MC species, respectively.^48^ Notably, the newly emerged absorption band of MC at 588 nm exhibits significant spectral overlap with the characteristic green emission of the Tb^3+^ center. As a result, the spectral overlap, close spatial proximity, and favorable dipole orientation between the Tb^3+^ and MC dual-emissive centers collectively provide an advantageous pathway for efficient FRET from the excited Tb^3+^ ions to the MC units (Fig. 3d).^38^ This efficient FRET process serves as the underlying mechanism enabling dynamic, light-controlled modulation of luminescence in these hybrid materials. We further evaluated the solid-state photochromic and photoluminescent responses of Poly-Tb(1–2). As shown in Fig. 3b and S13, the amorphous PMMA-based matrix offers sufficient conformational flexibility to enable partial ring-opening of SP moieties, thereby facilitating the rapid conversion between SP and MC even under solvent-free conditions.^33^ The initial photoluminescence spectrum recorded under 310 nm excitation exhibited a broad and weak emission band centered at 665 nm. This emission is characteristic of the MC state of spiropyran and is attributed to the high conformational freedom of the spiropyran moieties within the material, which allows partial photoisomerization of spiropyran from the closed-ring (SP) form to the open-ring (MC) form during the measurement.^36^ Upon prolonged UV exposure, a steady increase in the MC emission intensity was observed, accompanied by a gradual decrease in the intrinsic green emission of the Tb^3+^ center, indicating real-time modulation of energy transfer efficiency. The luminescence reached equilibrium within 80 seconds, demonstrating a rapid switching process. Concurrently, a clear and visually perceptible color transition occurred, from green to red under UV irradiation (Fig. 3c, S13b and SI Movie), visually validating the photochromic character of these metallopolymers. To further understand the FRET mechanism, time-resolved photoluminescence decay profiles of Tb^3+^ were measured under various UV irradiation durations (Fig. 3e, S14 and S15). A clear and progressive decrease in Tb^3+^ luminescence lifetime was recorded with increasing UV exposure time, confirming the progressive enhancement of FRET from the Tb^3+^ excited state to the MC acceptor. From the lifetime data, energy transfer efficiencies were calculated to be approximately 69% for Poly-Tb(1) and 37% for Poly-Tb(2), respectively. The variation in FRET performance is attributed to structural differences in the β-diketonate auxiliary ligands within the coordination environment, which influence not only the energy level alignment between the ligands and lanthanide ions but also the spatial proximity between the donor and acceptor, ultimately leading to distinct energy transfer efficiencies. The photoluminescence quantum yields (PLQYs) of the metallopolymers were further determined. Poly-Tb(1) exhibits a PLQY of 56.54%, while Poly-Tb(2) shows a PLQY of 45.85%, confirming the efficient luminescence performance of these systems (Table S2). Finally, we assessed the reversibility and fatigue resistance of the photochromic behavior of Poly-Tb(1–2) (Fig. S16 and S17). Upon irradiation with white light, the spiropyran moieties can revert from the open-ring (MC) state to the closed-ring (SP) state, restoring the initial green emission from the Tb^3+^ ions. Using Poly-Tb(1) as a model system, five consecutive photochromic switching cycles were conducted, showing slight degradation in emission intensity (Fig. 3f). This excellent cycle stability underscores the robustness and reversibility of the photo modulation process. Above all, these findings highlight the remarkable optical tunability, structural responsiveness, and long-term stability of these terbium-coordinated metallopolymers, making them promising candidates for the development of advanced smart luminescent devices, rewritable optical data storage, and multifunctional photonic materials.

### 2.3. pH-responsive behavior of Tb^3+^-metallopolymers

**Fig. 4:**
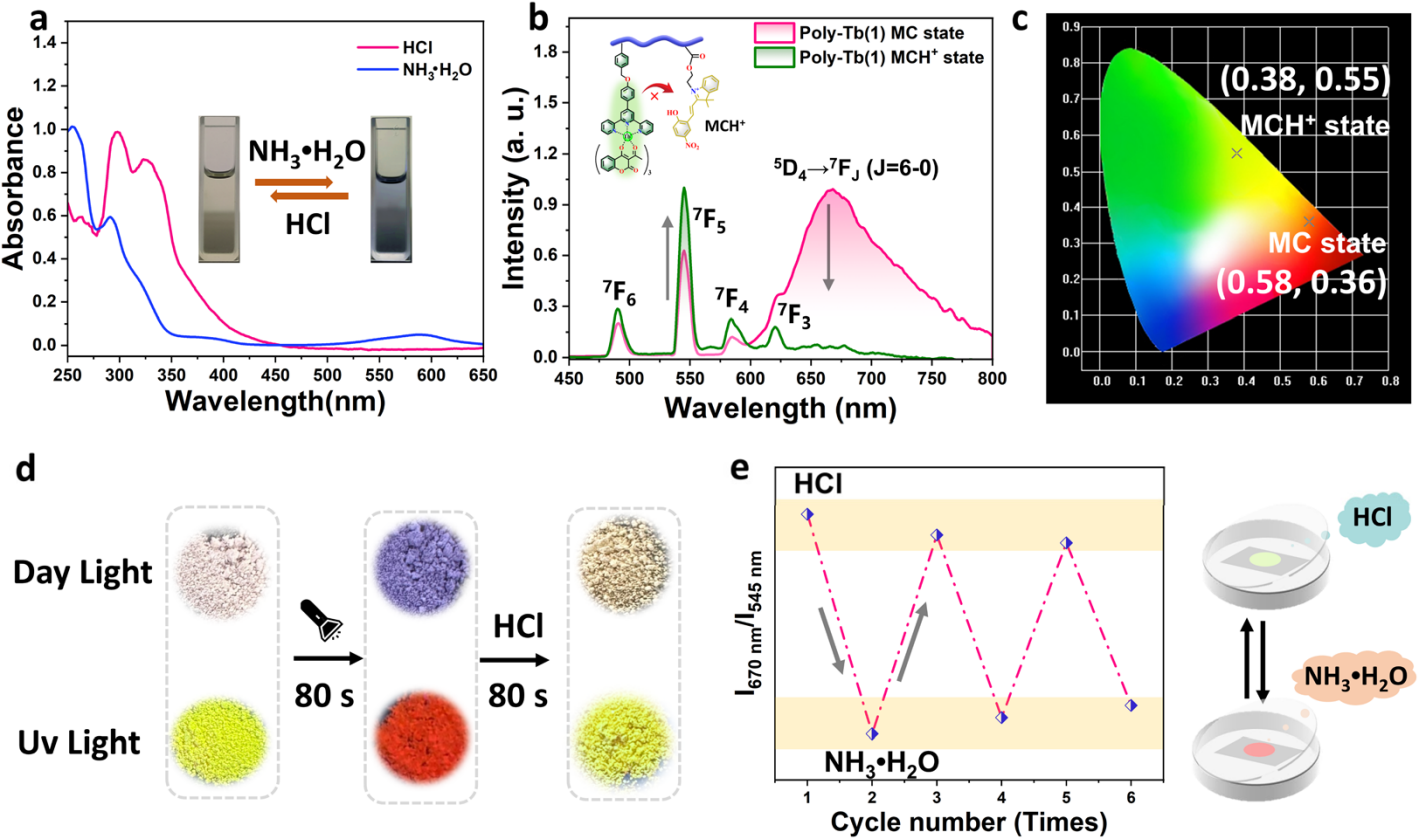
Acid/base stimuli-responsive behavior of Poly-Tb(1): (a) UV-vis absorption spectra of Poly-Tb(1) in solution under acidic (pH = 2) and basic (pH = 10) conditions; (b) emission spectra of solid-state Poly-Tb(1) under acid stimulation; (c) fluorescence color changes in response to acid stimulation; (d) luminescence photographs of Poly-Tb(1) under acid stimulation at 25 °C and under UV light; (e) reversibility of the acid/base stimuli-responsive behavior of Poly-Tb(1).

Spiropyran derivatives are well known not only for their photochromic behavior but also for their pronounced sensitivity to acid–base stimuli,^49,50^ which inspired us to further explore the pH-responsive luminescent properties of Poly-Tb(1–2). As illustrated in Fig. 4a and S18, UV-vis absorption spectra of Poly-Tb(1–2) solutions in THF (2 mg mL^−1^) demonstrate the disappearance of the characteristic MC absorption peak at 588 nm upon acidification. Crucially, this spectral change is fully reversible upon subsequent treatment with aqueous ammonia (NH_3_·H_2_O), confirming the robust and reversible acid–base-induced switching between the closed spiropyran (SP) and protonated merocyanine (MCH^+^) state in Poly-Tb(1–2). To further elucidate this phenomenon under solid-state conditions, Poly-Tb(1–2) (SP state) powders were exposed to hydrochloric acid vapor, and their photoluminescence (PL) responses were meticulously monitored. Interestingly, exposure of the SP form of Poly-Tb(1–2) to acidic vapor resulted in only minor reductions in the characteristic Tb^3+^ emission intensity after 3 minutes (Fig. S19). This contrasts markedly with typical reports of acid-induced quenching in lanthanide metallopolymers,^51^ suggesting that the β-diketonate ligands, together with the PMMA polymer matrix, provide an effective protective environment that significantly enhances the chemical durability and luminescence stability of the embedded Tb^3+^ ions under acidic conditions. In contrast, the Poly-Tb(1–2) (MC state) demonstrated a significantly different response to acidic stimuli. As shown in Fig. 4b and S20a, rapid quenching of MC emission from Poly-Tb(1–2) occurred within seconds of exposure to HCl vapor, coinciding with a simultaneous enhancement of Tb^3+^ luminescence. This behavior resulted in a visually distinct color transition from red to green for both Poly-Tb(1–2) (Fig. 4c, d and S20b), clearly demonstrating efficient acid-triggered luminescent switching. The underlying photophysical mechanism is clarified in Fig. S21, which illustrates the relative energy levels of the three spiropyran states, SP, MC, and protonated MC (MCH^+^), each possessing unique absorption and emission characteristics tunable by external stimuli. According to prior studies employing TD-DFT calculations,^52^ the excited-state energy levels of spiropyran derivatives SP, MC, and MCH^+^ have been established and are used here as reference values. The MC state generated under UV illumination exhibits a singlet excited-state energy of approximately 2.52 eV, which is slightly lower than that of the Tb^3+^ triplet excited state (20 500 cm^−1^, 2.54 eV). This energetic proximity enables efficient FRET from Tb^3+^ to MC, resulting in red emission. In contrast, the SP form, dominant under visible illumination, has a much higher S_1_ energy (3.19 eV), which prevents energy transfer and thus preserves the intrinsic green emission of Tb^3+^. Under acidic conditions, protonation converts MC into MCH^+^, elevating its S_1_ energy to 2.91 eV, above that of the Tb^3+^ excited state, thereby interrupting the FRET process and restoring green luminescence. Subsequent treatment with ammonia deprotonates MCH^+^, restoring the SP-to-MC photoisomerization capability of spiropyran. Upon UV irradiation, the sample exhibits a reversible luminescence change from green to red. To assess reversibility and practical durability, we conducted repeated acid–base cycling experiments on Poly-Tb(1). The material demonstrated nice luminescence switching stability over six successive HCl–NH_3_·H_2_O cycles (Fig. 4e), underscoring its robustness and potential for practical applications in externally triggered optical devices. Finally, to elucidate the role of Tb^3+^ ions in modulating the photochromic response, we examined the polymer backbone alone. As shown in Fig. S22, UV irradiation triggers a rapid fluorescence shift from orange-red to bright red, originating from the MC state of spiropyran. Upon HCl fumigation, the emission intensity decreases markedly. From these results, it is evident that in the absence of Tb^3+^ ions, the fluorescence color remains essentially unchanged, underscoring the critical contribution of Tb^3+^ ions to the photochromic process. Collectively, these results confirm that acid–base stimuli effectively modulate the luminescence properties of terbium-coordinated metallopolymers, establishing them as versatile and reliable platforms for sophisticated photophysical modulation.

### 2.4. Thermo-stimuli-responsive behavior of the Tb^3+^-metallopolymers

**Fig. 5:**
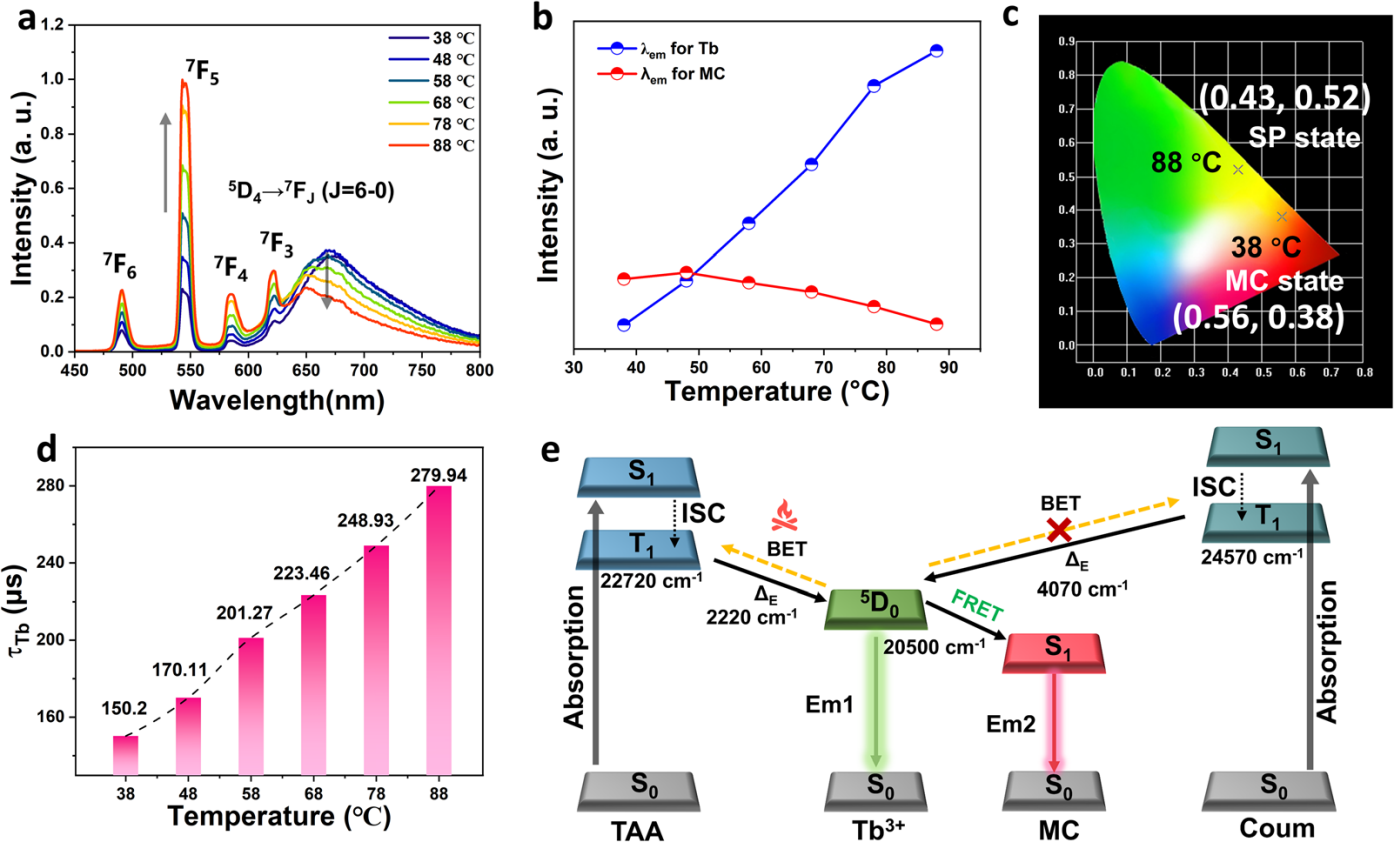
Thermoresponsive photoluminescence behavior of Poly-Tb(1): (a) emission spectra of Poly-Tb(1) in the MC state at different temperatures; (b) temperature-dependent emission intensity changes of Tb^3+^ and spiropyran moieties; (c) evolution of CIE chromaticity coordinates; (d) temperature-induced variation in the luminescence lifetime of Tb^3+^; (e) schematic illustration of the energy transfer process under thermal stimuli.

Besides responding to photo and pH stimuli, the reversible transformation between MC and SP forms of spiropyran also exhibits marked temperature sensitivity. Typically, elevated temperatures facilitate rapid thermal back-isomerization from the MC form to the SP state,^53^ suggesting potential thermochromic luminescence behaviors in terbium-coordinated metallopolymer. Driven by this hypothesis, we conducted comprehensive studies on the temperature-dependent luminescence properties of Poly-Tb(1–2). Intriguingly, Poly-Tb(1) and Poly-Tb(2) displayed markedly different thermal response behaviors. As depicted in Fig. S23, the emission intensities of both Tb^3+^ and MC significantly decreased in Poly-Tb(2) (MC state) as temperature increased, resulting in minimal residual emission from Tb^3+^ at 88 °C. Meanwhile, the luminescence color exhibited only slight changes before and after heating. In sharp contrast, Poly-Tb(1) (MC state) showed an unconventional negative thermal quenching phenomenon,^54^ in which Tb^3+^ emission intensity notably increased with rising temperature (Fig. 5a and b), while MC emission from the spiropyran unit steadily diminished. Consequently, the emission color of Poly-Tb(1) visibly transitioned from red back to green upon heating (Fig. 5c and SI Movie), indicating distinct thermochromic luminescence characteristics. Given that lanthanide complexes commonly exhibit decreased luminescence intensity at elevated temperatures due to thermal quenching, the anomalous thermal enhancement observed in Poly-Tb(1) is particularly remarkable. We hypothesized that thermal back-isomerization from the MC to the SP form at elevated temperatures suppresses the energy transfer from Tb^3+^ to MC, thereby enhancing Tb^3+^ emission efficiency. To validate this, we measured the luminescence lifetimes of Tb^3+^ centers at different temperatures (Fig. 5d and S24). Indeed, the Tb^3+^ lifetime in Poly-Tb(1) (MC state) markedly increased from 150.2 μs at 38 °C to 279.94 μs at 88 °C, confirming suppression of non-radiative relaxation pathways. Conversely, Poly-Tb(2) (MC state) exhibited significant lifetime reduction under identical conditions (Fig. S25), confirming severe thermal quenching. Further investigations into ligand-to-metal energy transfer pathways provided mechanistic insights into these divergent behaviors (Fig. 5e). Previous studies have demonstrated that the triplet energy levels of coordinating ligands significantly influence the sensitization efficiency of Tb^3+^ emissions.^55^ For instance, the triplet level of TAA is 22 720 cm^−1^,^56^ yielding a relatively small energy gap of only 2220 cm^−1^ with respect to the Tb^3+^ emitting level. This narrow gap facilitates thermally activated back energy transfer at elevated temperatures, resulting in pronounced luminescence quenching.^57^ In contrast, coumarin-based ligands possess a higher triplet energy level of 24 570 cm^−1^,^58^ corresponding to a larger energy gap of 4070 cm^−1^. This energetically favorable alignment effectively suppresses thermally activated back energy transfer pathways, thereby enhancing the thermal stability of Tb^3+^ luminescence. Accordingly, upon heating, the thermally induced isomerization of MC to SP rapidly interrupts the FRET process from Tb^3+^ to MC in Poly-Tb(1). Benefiting from the large energy gap between the β-diketone ligand and the Tb^3+^ ion, the luminescence of Tb^3+^ is minimally affected by thermal back-transfer, resulting in a thermally enhanced emission behavior. In contrast, although the MC-to-SP transition in Poly-Tb(2) also blocks the FRET channel, the smaller energy gap between the ligand TAA and Tb^3+^ facilitates back energy transfer at elevated temperatures, leading to rapid luminescence quenching. As a result, the emission intensity of Poly-Tb(2) decreases with increasing temperature. We further evaluated the temperature-dependent emission stability of the reference metallopolymer systems Poly-Tb(R1–R2) (Fig. S26). Poly-Tb(R1) exhibited excellent thermal emission stability with only minimal Tb^3+^ emission reduction at elevated temperatures. In contrast, Poly-Tb(R2) showed nearly complete luminescence quenching under identical conditions. These observations reinforce the critical role of precise ligand selection and energy level alignment in effectively modulating the thermo-responsive photophysical properties of terbium-coordinated metallopolymers.

### 2.5. Stimuli-driven anticounterfeiting and sequential logic encryption

**Fig. 6:**
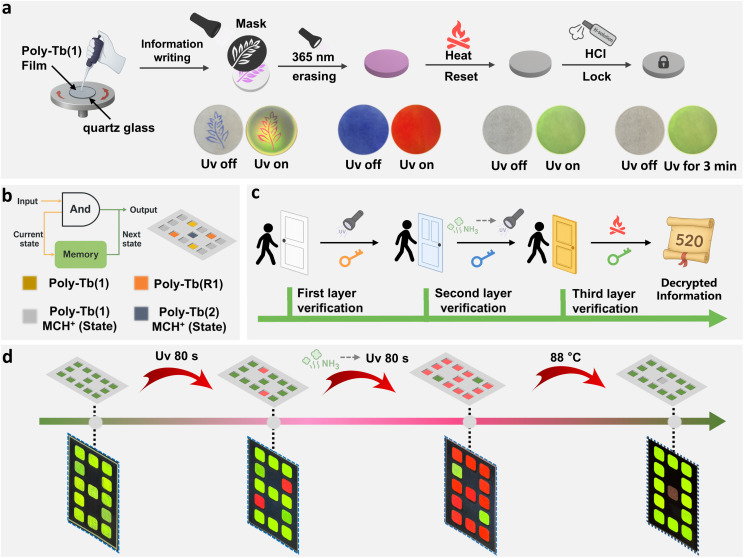
(a) Photolithography patterning and multi-stimuli responsive characteristics of the Poly-Tb(1) film; (b and c) schematic illustrations of sequential stimulus-responsive logic gates and multi-level information encryption devices based on lanthanide metallopolymers; (d) multi-level information encryption applications of lanthanide metallopolymers.

Considering the excellent multi-stimuli responsiveness and cycling stability of the developed lanthanide metallopolymers, we further explored their practical application potential. Initially, a thin optical anticounterfeiting film was prepared by spin-coating a dichloromethane solution of Poly-Tb(1) onto a quartz substrate (Fig. 6a). We then demonstrated the capability of this film for reversible information writing and erasing. By applying a mask and exposing the film to UV light, a leaf-shaped encrypted pattern could be easily inscribed. Remarkably, the pattern exhibited a blue–purple appearance under daylight and a distinct red fluorescence under UV illumination. Subsequent extended UV exposure efficiently erased the written pattern, turning the entire film uniformly blue–purple under ambient conditions. Notably, heating the film to 88 °C effectively restored it to its original pristine state. Finally, spraying the film surface with an ethanol solution containing hydrochloric acid effectively locked its photochromic behavior, rendering the pattern stable and unresponsive even after prolonged UV irradiation of up to three minutes. Inspired by the multi-stimuli responsive fluorescence behaviors described above, we further explored the potential of these lanthanide metallopolymers in practical applications for sequential logic encryption (SLE).^59–61^ Unlike conventional logic devices, sequential logic gates are a distinct class of circuits whose outputs depend not only on current inputs but also on the historical sequence of inputs. Their core attributes—memory capability and feedback mechanisms—enable dynamic storage and iterative updating of input information over time. As depicted in Fig. 6b, taking a three-input sequential logic gate as an illustrative example, the system produces a valid output state of “1” only when a specific sequence of inputs is applied in the prescribed order.^62^ Any deviation in the input order or combination results in an output of “0”, mimicking the operation of keypad lock mechanisms commonly used in daily life. Encryption architectures based on such SLE strategies, therefore, necessitate strict adherence to the designated input sequence and leverage deceptive feedback pathways to robustly deter unauthorized decryption attempts.^63^ We utilized the tunable multicolor fluorescence properties of terbium-coordinated metallopolymers, employing sequential combinations of light, alkaline gases, and heat as decoding keys, significantly enhancing the overall security of the encryption system. Specifically, Poly-Tb(1), Poly-Tb(R1), and Poly-Tb(1–2) (MCH^+^) were integrated as individual pixels within a quartz substrate-based digital matrix. Unlike conventional encryption methods relying on single-stimulus or simultaneous multiple stimuli responses,^64,65^ our encryption strategy employs a sequential logic-based system, meaning successful decryption strictly depends on a predetermined order of multiple stimuli rather than arbitrary combinations. Initially, UV illumination of the digital matrix yields a green fluorescent display forming the digit “8”. Upon continued UV exposure, pixels composed of embedded Poly-Tb(1) transform from green to red, changing the displayed digit to “5”. Next, exposure to ammonia vapor unlocks the previously stimulus-locked Poly-Tb(1–2) (MCH^+^) pixels, which subsequently turn red after further UV irradiation, revealing the digit “2”. Finally, upon heating the matrix, the emission from Poly-Tb(2) is substantially quenched due to the inherent thermal sensitivity of lanthanide-based luminescence. Simultaneously, the red-emissive Poly-Tb(1) in its merocyanine (MC) state thermally reverts to the non-emissive spiropyran (SP) form, which subsequently recovers the characteristic green Tb^3+^ emission. As a result, the collective emission pattern shifts to display the digit “0”. Importantly, this encryption system is highly programmable, with the encrypted information determined by the strategic embedding of specific pixel materials. The output relies critically on the sequence of stimuli applied, ensuring robust resistance against brute-force decoding attempts. Moreover, Poly-Tb(1–2) (MCH^+^) pixels inherently possess an information-erasure mechanism activated by alkaline vapor exposure, meaning any incorrect stimulus sequence or even a single reading can irreversibly erase the stored information, providing additional security akin to a self-destructing encryption mechanism.

## 3 Conclusions

In summary, we have successfully designed and synthesized a novel class of luminescent materials exhibiting reversible fluorescence responses to multiple external stimuli, including light, temperature and pH, by covalently incorporating spiropyran and Tb^3+^-based complexes into polymeric frameworks. These materials preserve the intrinsic multi-stimuli-responsive properties of spiropyran, exhibiting rapid and reversible solid-state photochromism. Through effective Förster resonance energy transfer between Tb^3+^ ions and spiropyran moieties, significant emission color shifts were achieved under different external stimuli. Notably, by precisely tuning the ligand energy levels around the Tb^3+^ ions, we demonstrated the controlled manipulation of intramolecular energy transfer pathways within the metallopolymer system. This enabled the realization of the unusual anti-thermal quenching behavior of Tb^3+^ ions, resulting in reversible thermochromic luminescence. Capitalizing on these unique multi-responsive characteristics, we developed a programmable encryption–decryption strategy based on the sequential application of various stimuli. This study not only provides a novel route for engineering stimulus-responsive lanthanide metallopolymers but also offers valuable theoretical insights and practical strategies for advancing the design of next-generation photo-responsive luminescent materials.

## Author contributions

W. X. Feng conceived the research plan and wrote the initial draft of the manuscript. S. M. Lu and X. L. Liao carried out the sample preparation and characterization. D. Han, Q. R. Guo, and Y. Zhao contributed to figure preparation and data analysis. W. Tian and H. X. Yan revised and improved the manuscript.

## Conflicts of interest

There are no conflicts to declare.

## Supplementary Material

SC-OLF-D5SC06759F-s001

SC-OLF-D5SC06759F-s002

## Data Availability

The data supporting this article have been included as part of the supplementary information (SI). Supplementary information: additional experimental details and materials characterization data (*e.g.*, NMR, PL, and TGA). See DOI: https://doi.org/10.1039/d5sc06759f.
