# Supplementary material for: Harnessing spiropyran isomerization in lanthanide metallopolymers for sequential logic encryption and anticounterfeiting
Source: Chem Sci. 2025 Oct 27;16(47):22517–26. doi: 10.1039/d5sc06759f (PMC12556595; doi:10.1039/d5sc06759f)
Supplement: SC-016-D5SC06759F-s002 [file SC-016-D5SC06759F-s002.pdf]

Supplementary Information (SI) for Chemical Science.
This journal is © The Royal Society of Chemistry 2025

Supporting Information

Harnessing Spiropyran Isomerization in Lanthanide Metallopolymers for Sequential Logic Encryption and Anticounterfeiting

Weixu Feng, Xiaolin Liao, Sumin Lu, Dong Han, Qianrong Guo, Yan Zhao, Wei Tian and Hongxia Yan*

Xi'an Key Laboratory of Hybrid Luminescent Materials and Photonic Device, School of Chemistry and Chemical engineering, Northwestern Polytechnical University, Xi'an 710129, Shaanxi, China.

*Email: hongxiayan@nwpu.edu.cn (H. Y)

Table of Contents

Materials and Methods……S4

Synthesis of 4'-(4-((4-vinylbenzyl)oxy)phenyl)-2,2':6',2''-terpyridine (VTPY)……S5

Fig. S1 ^{1}H NMR spectra of VTPY ($CDCl_3$, room temperature)……S6

Synthesis of SP……S6

Fig. S2 ^{1}H NMR spectra of SP ($CDCl_3$, room temperature)……S7

Fig. S3 ^{13}C NMR spectra of SP ($CDCl_3$, room temperature)……S8

Synthesis of the polymer backbone Poly(VTPY-*co*-SP-*co*-MMA)……S8

Synthesis of 3-acetyl-4-hydroxy-coumarin (Coum)……S9

Fig. S4 ^{1}H NMR spectra of Coum ($CDCl_3$, room temperature)……S9

Fig. S5 ^{13}C NMR spectra of Coum ($CDCl_3$, room temperature)……S10

Synthesis of $Tb(Coum)_3·2H_2O$……S10

Synthesis of the Poly-Tb(1)……S10

Synthesis of Poly-Tb(2)……S11

Synthesis of the reference polymer backbone Poly(VTPY-*co*-MMA) (Poly(R))……S11

Scheme S1. Synthetic pathway for Poly(R) and Poly-Tb(R1-2)……S12

Synthesis of Poly-Tb(R1)……S12

Synthesis of Poly-Tb(R2)……S13

Fig. S6 FT-IR spectrum of Poly and Poly-Tb(2)……S13

Fig. S7. PXRD patterns of Poly and Poly-Tb(1-2)……S14

Fig. S8 GPC curves of Poly-Tb(1-2)……S14

Table S1. GPC data for Poly-Tb(1) and Poly-Tb(2)……S14

Fig. S9 XPS spectra of Poly……S15

Fig. S10 XPS spectra of Poly-Tb(2)……S15

Fig. S11 TGA curves of Poly-Tb(1) and Poly-Tb(2) Poly-Tb(1)……S15

Fig. S12 UV-Vis absorption spectra of Poly-Tb(2) under different durations of UV irradiation…… S16

Fig. S13 (a) Photoluminescence (PL) spectra of Poly-Tb(2) recorded after different durations of UV irradiation and (b) corresponding CIE chromaticity coordinates……S16

Fig. S14 Luminescence lifetime decay profiles of Tb^{3+} emission from Poly-Tb(1) under different UV irradiation durations (λ_{ex} = 310 nm)……S17

Fig. S15 Luminescence lifetime decay profiles of Tb^{3+} emission from Poly-Tb(2) under different UV irradiation durations (λ_{ex} = 310 nm)……S17

Table S2 Photoluminescence Quantum Yields of Poly-Tb(1) and Poly-Tb(2)……S17

Fig. S16 Photoluminescence spectra of Poly-Tb(1) recorded after different durations of white light irradiation and corresponding CIE chromaticity coordinates……S18

Fig. S17 (a) Photoluminescence (PL) spectra of Poly-Tb(2) recorded after different durations of white light irradiation and (b) corresponding CIE chromaticity coordinates………S18
Fig. S18 UV-vis absorption spectra of Poly-Tb(1) in solution under acidic and basic conditions………S19
Fig. S19 Emission spectra of (a) Poly-Tb(1) and (b) Poly-Tb(2) in the SP state after fuming with concentration HCl for different durations……………………………………………………………S19
Fig. S20 Emission spectra of Poly-Tb(2) in the MC state after fuming with conc. HCl……………S19
Fig. S21 Simplified Jablonski diagram to explain the Förster energy transfer process between the donor and acceptor in the lanthanide metallopolymer……………………………………………S20
Fig. S22 Emission spectra and fluorescence color changes of the polymer backbone (Poly) under UV light and acid/base stimuli (λ_{em} = 310 nm)…………………………………………………S20
Fig. S23 (a) Photoluminescence (PL) spectra of Poly-Tb(2) (SP state) recorded under different temperature and (b) corresponding CIE chromaticity coordinates…………………………………S20
Fig. S24 Luminescence lifetime decay profiles of Tb^{3+} emission from Poly-Tb(1) (MC state) under different temperatures (λ_{ex} = 310 nm)……………………………………………………………S21
Fig. S25 Luminescence lifetime decay profiles of Poly-Tb(2) under different temperatures………S21
Fig. S26 Photoluminescence (PL) spectra of (a) Poly-Tb(R1) and (b) Poly-Tb(R2) recorded under different temperatures……………………………………………………………………………………………S22
References………S22

Materials and Methods

All chemical reagents utilized for the organic synthesis were of reagent grade and sourced commercially, with no additional purification steps unless otherwise noted. Tetrahydrofuran (THF), suitable for HPLC applications, was obtained from Fisher Scientific, while other solvents were procured from Sigma Aldrich and used as received. Azobis(isobutyronitrile) (AIBN) underwent recrystallization twice in ethanol prior to use to ensure its purity. Reactions involving moisture- or oxygen-sensitive species were conducted under inert atmosphere using standard Schlenk techniques in strictly anhydrous and oxygen-free environments. Fourier-transform infrared (FTIR) spectra were recorded on a WQF-310 spectrometer in the range of 4000 to 500 cm^{-1}. ^{1}H NMR spectra were acquired using a Bruker Plus 400 MHz spectrometer in deuterated chloroform ($CDCl_3$), with tetramethylsilane (TMS) as the internal reference at ambient temperature. X-ray photoelectron spectroscopy (XPS) analysis was performed on a PHI 5700 instrument, equipped with both monochromatic Al Kα and dual Mg X-ray sources, featuring depth profiling and angle-resolved detection capabilities. UV-Vis absorption measurements were conducted using a Shimadzu UV-3159 spectrophotometer, while Photoluminescence lifetimes and temperature-dependent PL spectra were measured using an Edinburgh Instruments FLS-1000 spectrometer. Molecular mass and polydispersity indices were evaluated through gel permeation chromatography (GPC), utilizing a Waters 1525 binary pump system coupled with a Waters 2414 refractive index detector, with THF as the mobile phase and 10 µm particle size columns from American Polymer Standards, Molar mass averages and dispersity were determined using a calibration curve obtained from polystyrene standards (Mp = 1.0–1000 kDa). The retention times of the samples were converted to molar mass values according to this calibration, and Mn, Mw,

and Đ were calculated from the dRI detector response. The CIE chromaticity coordinates of samples were computed according to the standards set by the International Commission on Illumination (CIE). The photo-stimuli response experiments were performed using a 5 W 365 nm UV lamp positioned at a fixed distance of 10 cm from the sample, while the pH-responsive experiments were conducted in a sealed environment by placing a drop of concentrated hydrochloric acid or ammonia solution adjacent to the sample, the temperature-responsive experiments were performed using a thermostatic heating stage. The FRET efficiency (E) was quantitatively evaluated by comparing the fluorescence lifetimes of the donor in the absence (τ_D) and presence (τ_{DA}) of the acceptor, according to the following equation:[S1]

$$E = 1 - \tau_{DA}/\tau_D$$

where, τ_D represents the intrinsic excited-state lifetime of the Tb^{3+} in the presence of the closed-ring spiropyran (SP) form, while τ_{DA} corresponds to the Tb^{3+} lifetime when energy transfer occurs to the merocyanine (MC) form upon ring-opening.

Synthesis of 4'-(4-((4-vinylbenzyl)oxy)phenyl)-2,2':6',2''-terpyridine (VTPY)

The synthetic route of VTPY was carried out according to a previously reported method.[S2] Firstly, the intermediate TPY-OH was prepared. 2-Acetylpyridine (2.42 g, 20 mmol) and p-hydroxybenzaldehyde (1.22 g, 10 mmol) were placed in a 100 mL round-bottom flask and dissolved in 16 mL of ethanol. Potassium hydroxide (1.21 g, 21 mmol) was then added, followed by aqueous ammonia ($NH_3 \cdot H_2O$). The reaction mixture was stirred at room temperature for 24 hours. During the reaction, the solution gradually turned reddish-brown. Upon completion, glacial acetic acid was slowly added until no further precipitate formed. The precipitate was washed three times with anhydrous ethanol and dried to constant weight, yielding a crude product of 1.56 g (25% yield). ^1H

NMR (400 MHz, DMSO-d_6) δ (ppm): 9.94 (s, 1H), 8.76 (d, J = 4.3 Hz, 2H), 8.67 (d, J = 6.3 Hz, 4H), 8.04 (t, J = 7.0 Hz, 2H), 7.80 (d, J = 8.5 Hz, 2H), 7.55-7.51 (m, 2H), 6.98 (d, J = 8.5 Hz, 2H).

Subsequently, TPY-OH (0.24 g, 0.74 mmol) and 4-chloromethylstyrene (0.22 g, 1.44 mmol) were dissolved in 10 mL of DMF in a 100 mL round-bottom flask. Potassium hydroxide (0.08 g, 1.45 mmol) was added, and the reaction mixture was stirred at room temperature for 24 hours. The solution color changed from red to yellow over time. After completion, distilled water was added until no precipitate formed. The solid product was collected by filtration, washed with anhydrous ethanol, and dried at a constant temperature to yield 0.23 g of product (70% yield). ^{1}H NMR (400 MHz, $CDCl_3$) δ (ppm): 8.75-8.65 (m, 6H), 7.91-7.85 (m, 4H), 7.44 (d, J = 3.2 Hz, 4H), 7.37-7.33 (m, 2H), 7.10 (d, J = 8.7 Hz, 2H), 6.78-6.71 (m, 1H), 5.78 (d, J = 17.6 Hz, 1H), 5.27 (d, J = 10.9 Hz, 1H), 5.13 (s, 2H).

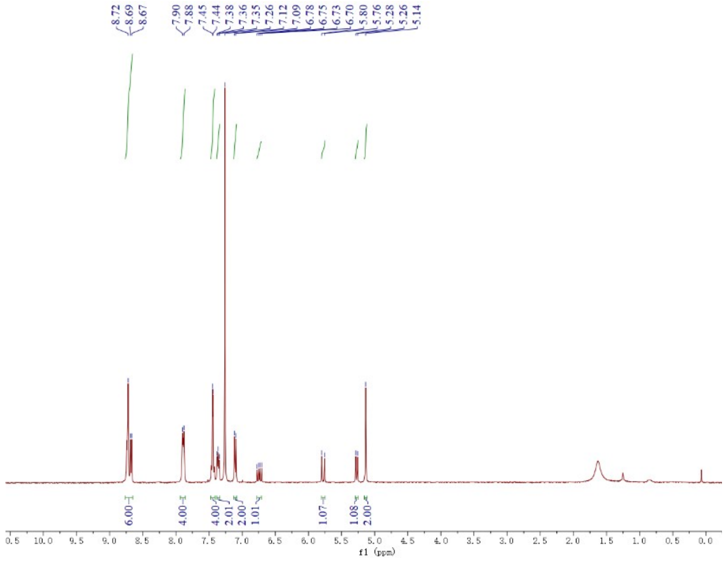

Fig. S1 ^{1}H NMR spectra of VTPY ($CDCl_3$, room temperature).

Synthesis of 2-(3',3'-dimethyl-6-nitrospiro[chromene-2,2'-indolin]-1'-yl)ethyl methacrylate (SP)

The synthetic route for SP was carried out according to a previously reported method.[S3] In a 100 mL round-bottom flask, dicyclohexylcarbodiimide (DCC, 1.8 g, 8.74 mmol), 4-

dimethylaminopyridine (DMAP, 0.05 g, 0.41 mmol), and SP-OH (3.0 g, 8.5 mmol) were dissolved in 30 mL of dichloromethane. The reaction mixture was stirred in an ice-water bath under a nitrogen atmosphere for 30 minutes. Methyl methacrylate (0.75 g, 8.72 mmol) was then added, and stirring was continued for another 30 minutes in the ice bath. The reaction was then allowed to proceed at room temperature for an additional 48 hours under nitrogen. Upon completion, a large amount of precipitate was observed. The reaction mixture was filtered, and the filtrate was concentrated under reduced pressure. The crude product was purified by column chromatography using ethyl acetate/petroleum ether (v/v = 1:4) as the eluent. After drying under constant temperature, a pale-yellow powder was obtained (0.57 g, yield: 47.4%). ^{1}H NMR (400 MHz, $CDCl_3$) δ (ppm): 8.01 (d, J = 10.2 Hz, 2H), 7.21 (t, 1H), 7.09 (d, 1H), 6.93-6.87 (m, 2H), 6.73 (dd, 2H), 6.07 (s, 1H), 5.87 (d, 1H), 5.56 (s, 1H), 4.30 (t, 2H), 3.60-3.38 (m, 2H), 1.92 (s, 3H), 1.28 (s, 3H), 1.16 (s, 3H). ^{13}C NMR (126 MHz, $CDCl_3$) δ (ppm): 136.09, 135.74, 128.34, 127.89, 126.02, 125.94, 122.82, 121.83, 119.98, 118.46, 115.61, 106.80, 106.56, 62.67, 52.85, 42.47, 30.81, 26.95, 25.88, 19.88, 18.39.

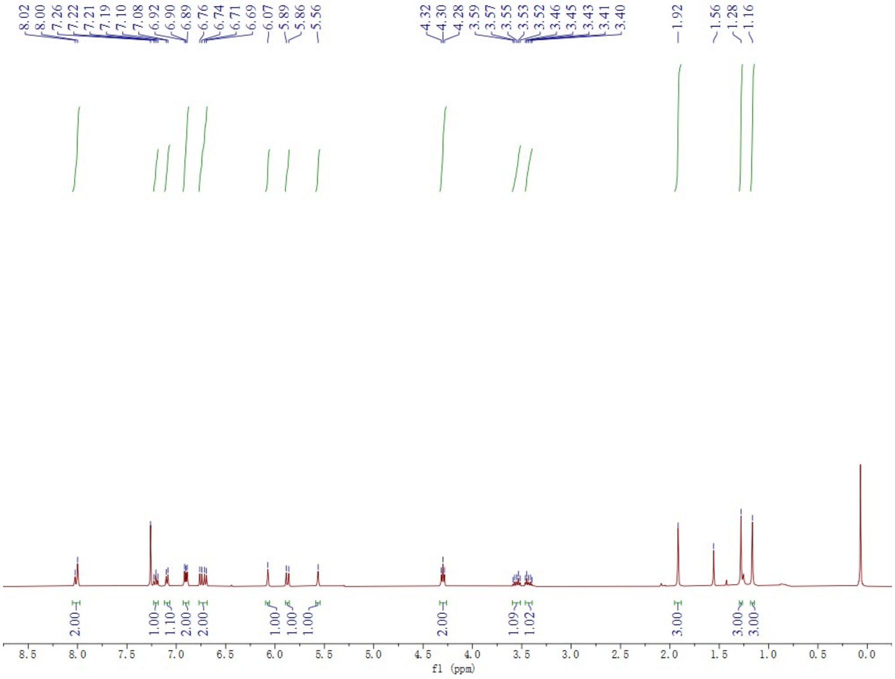

Fig. S2 ^{1}H NMR spectra of SP ($CDCl_3$, room temperature).

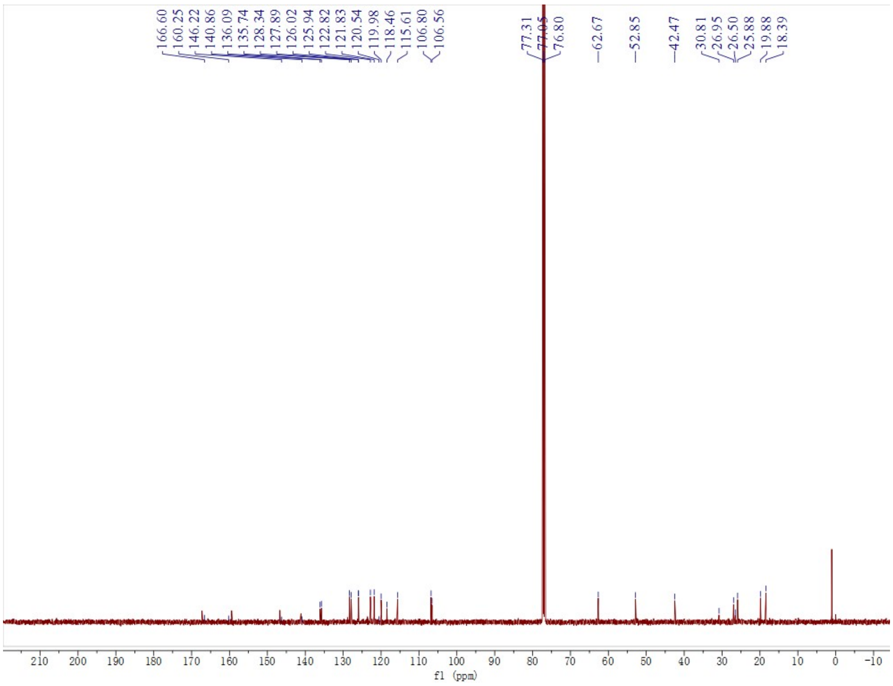

Fig. S3 ^{13}C NMR spectra of SP ($CDCl_3$, room temperature).

Synthesis of the polymer backbone Poly(VTPY-*co*-SP-*co*-MMA)

VTPY (44.1 mg, 0.1 mmol), SP (170.6 mg, 0.4 mmol), and MMA (1.0 g, 10 mmol) were added to a 100 mL round-bottom flask at a molar ratio of 1:4:100. The monomers were completely dissolved in DMF, and under a nitrogen atmosphere, AIBN (1.5 mol%) was added as the initiator. The reaction mixture was gradually heated to 80 °C for 48 hours. Upon completion, a clear solution was obtained. After dilution with dichloromethane, the mixture was poured into ice-cold anhydrous methanol to precipitate a pale-yellow solid. The precipitate was collected and dried to constant weight using a vacuum freeze dryer to afford a pale-yellow powder of Poly(VTPY-*co*-SP-*co*-MMA) in 45% yield. ^{1}H NMR (400 MHz, $CDCl_3$) δ (ppm): 8.70 (s, 6H), 8.04 (s, 4H), 7.88 (s, 6H), 7.35 (s, 6H), 7.09-6.95 (m, 14H), 6.77 (s, 4H), 5.07 (s, 2H), 4.07 (s, 6H), 3.69 (s, 216H), 1.81 (s, 140H), 1.01 (s, 60H), 0.82 (s, 150H). FT-IR (KBr, cm^{-1}): 2993 (w), 2948 (w), 1727 (vs), 1601 (w), 1585 (w), 1513 (w), 1435 (w), 1387 (w), 1240 (m), 1147 (vs), 989 (w), 840 (m), 795 (vs), 750 (w).

Synthesis of 3-acetyl-4-hydroxy-coumarin (Coum)

The Coum was synthesized according to a previously reported method.[S4] 4-hydroxy coumarine (3.0 g, 18.6 mmol) was dissolved in 20 mL of acetic acid in a 100 mL round-bottom flask. Phosphoryl chloride ($POCl_3$, 5.6 mL) was then added, and the mixture was refluxed at 117-119 °C for 30 minutes. An additional 1 mL of $POCl_3$ was added, and the reaction was continued under reflux for another 30 minutes. A large amount of precipitate was formed. After filtration, an appropriate amount of ethanol was added to the filtrate to obtain a clear solution. Upon standing for three days, a white solid precipitated. The product was collected by filtration and dried at 45 °C to yield a white powder in 72% yield. ^{1}H NMR (400 MHz, $CDCl_3$) δ (ppm): 8.07 (dd, J = 8.0, 1.8 Hz, 1H), 7.73-7.66 (m, 1H), 7.33 (dd, J = 17.6, 8.2 Hz, 2H), 2.79 (s, 3H). ^{13}C NMR (101 MHz, $CDCl_3$) δ (ppm): 206.09, 178.73, 160.09, 154.77, 136.17, 125.67, 124.47, 117.06, 115.27, 101.43, 30.13.

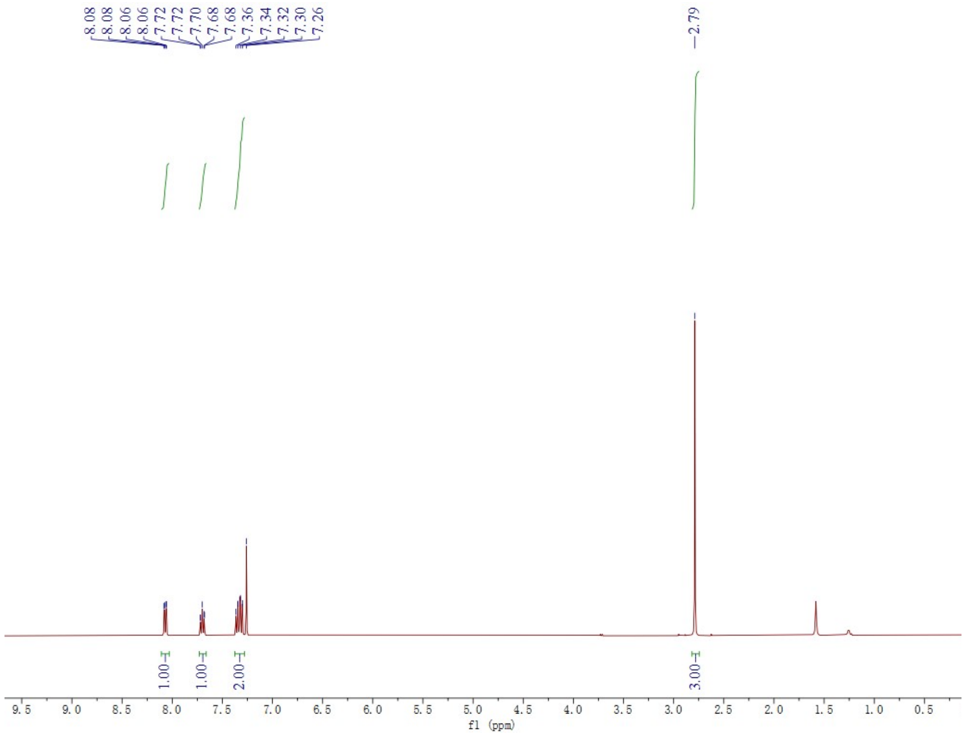

Fig. S4. ^{1}H NMR spectra of Coum ($CDCl_3$, room temperature).

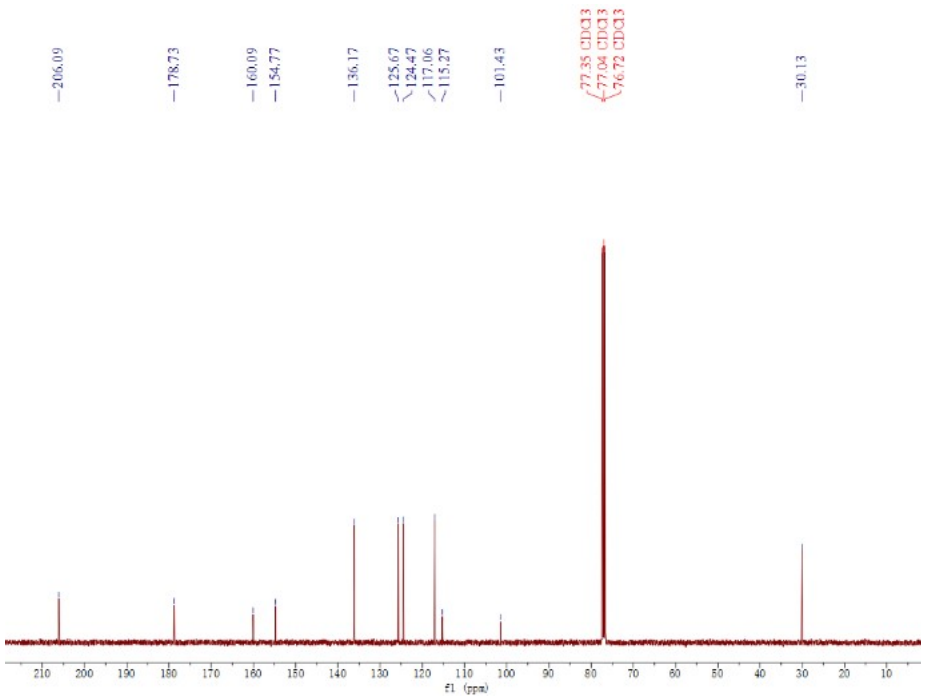

Fig. S5. ^{13}C NMR spectra of Coum ($CDCl_3$, room temperature).

Synthesis of Tb(Coum)$_3$·2H$_2$O

Coum (0.918 g, 4.5 mmol) was dissolved in 5 mL of anhydrous ethanol. Separately, terbium(III) chloride hexahydrate (0.56 g, 1.5 mmol) was completely dissolved in deionized water and transferred into a 50 mL round-bottom flask. The pH of the solution was adjusted to 6 using aqueous NaOH. The mixture was then heated to 60 °C and stirred for 6 hours. A large amount of precipitate formed during the reaction. After filtration, the solid was dried at constant temperature to afford a white powder (0.66 g), with a yield of 65.3%. FT-IR (KBr, cm^{-1}): 3418 (vs), 3076 (w), 1715 (m), 1607 (w), 1547 (w), 1489 (w), 1424 (w), 1360 (w), 1248 (w), 1203 (w), 1158 (w), 1106 (w), 1027 (vs), 898 (w), 753 (m), 669 (w).

Synthesis of Poly-Tb(1)

Poly(VTPY-*co*-SP-*co*-MMA) (220 mg) and Tb(Coum)$_3$•2H$_2$O (0.02 mmol, 16 mg) were added to a 100 mL round-bottom flask, followed by the addition of 10 mL of tetrahydrofuran (THF). The mixture was stirred until fully dissolved, yielding a clear and transparent solution. The solution was

then gradually heated to 60 °C for 6 hours. Upon completion of the reaction, *n*-hexane was added to induce precipitation, affording a white solid. The precipitate was collected by filtration and dried at 45 °C to obtain a white powder, identified as Poly-Tb(1), with a yield of 87%. FT-IR (KBr, cm^{-1}): 2996 (w), 2948 (w), 1729 (vs), 1614 (w), 1585 (w), 1435 (w), 1389 (w), 1241 (w), 1149 (w), 989 (w), 840 (w), 752 (w).

Synthesis of the Poly-Tb(2)

Poly(VTPY-*co*-SP-*co*-MMA) (220 mg) and $Tb(TAA)_3{\cdot}2H_2O$ (0.02 mmol, 13 mg) were added to a 100 mL round-bottom flask and dissolved in 15 mL of tetrahydrofuran (THF). The solution was gradually heated to 60 °C for 6 hours. Upon completion of the reaction, n-hexane was added to induce precipitation, yielding a white solid. The precipitate was collected by filtration and dried at a constant temperature to afford Poly-Tb(2) as a white powder in 86% yield. FT-IR (KBr, cm^{-1}): 2993 (w), 2948 (w), 1727 (vs), 1513 (w), 1435 (w), 1387 (w), 1240 (m), 1147 (vs), 989 (w), 840 (m), 750 (w).

Synthesis of the reference polymer backbone Poly(VTPY-*co*-MMA) (Poly(R))

VTPY (44.1 mg, 0.1 mmol) and MMA (1.0 g, 10 mmol) were added to a 100 mL round-bottom flask at a molar ratio of 1:100. The monomers were completely dissolved in DMF, and under a nitrogen atmosphere, AIBN (1.5 mol%) was added as the initiator. The reaction mixture was gradually heated to 80 °C for 48 hours. Upon completion, a clear solution was obtained. After dilution with dichloromethane, the mixture was poured into ice-cold anhydrous methanol to precipitate a pale-yellow solid. The precipitate was collected and dried to constant

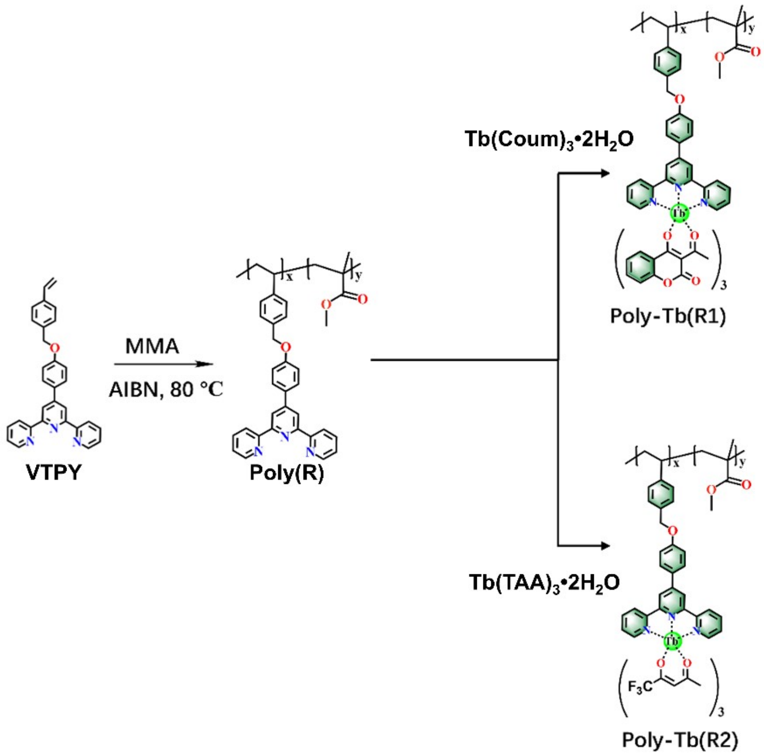

Scheme S1. Synthetic pathway for Poly(R) and Poly-Tb(R1-2).

weight using a vacuum freeze dryer to afford a pale-yellow powder of Poly(VTPY-*co*-MMA) in 45% yield. ^1H NMR (400 MHz, $CDCl_3$) δ (ppm): 8.75 - 8.64 (m, 6H), 7.88 (d, 4H), 7.36 (s, 6H), 7.00 (s, 2H), 5.00 (s, 2H), 3.68-3.53 (m, 210H), 1.74 (s, 140H), 1.18 (s, 55H), 0.95 (s, 55H), 0.76 (s, 55H). FT-IR (KBr, cm^{-1}): 2966 (w), 1724 (vs), 1687 (w), 1649 (w), 1579 (w), 1513 (w), 1448 (w), 1391 (w), 1274 (m), 1237 (w), 1186 (w), 1143 (vs), 1064 (m), 979 (w), 857 (m), 805 (w), 754 (w).

Synthesis of Poly-Tb(R1)

Poly(VTPY-*co*-MMA) (220 mg) and $Tb(Coum)_3$•2H2O (0.02 mmol, 16 mg) were added to a 100 mL round-bottom flask, followed by the addition of 5 mL of tetrahydrofuran (THF). The mixture was stirred until fully dissolved, yielding a clear and transparent solution. The solution was then gradually heated to 60 °C for 6 hours. Upon completion of the reaction, n-hexane was added to induce precipitation, affording a white solid. The precipitate was collected by filtration and dried at 45 °C to obtain a white powder, identified as Poly-Tb(R), with a yield of 85%. FT-IR (KBr, cm^{-1}):

2932 (w), 2857 (w), 1729 (vs), 1603 (w), 1547 (w), 1490 (w), 1440 (w), 1265 (m), 1239 (m), 1190 (m), 1145 (w), 1064 (m), 989 (w), 839 (w), 751 (w).

Synthesis of Poly-Tb(R2)

Poly(VTPY-*co*-MMA) (220 mg) and $Tb(TAA)_3$•2H2O (0.02 mmol, 13 mg) were added to a 100 mL round-bottom flask, followed by the addition of 5 mL of tetrahydrofuran (THF). The mixture was stirred until fully dissolved, yielding a clear and transparent solution. The solution was then gradually heated to 60 °C for 6 hours. Upon completion of the reaction, n-hexane was added to induce precipitation, affording a white solid. The precipitate was collected by filtration and dried at 45 °C to obtain a white powder, identified as Poly-Tb(R2), with a yield of 81%. FT-IR (KBr, cm^{-1}): 2949 (s), 2845 (w), 1729 (vs), 1637 (w), 1605(w), 1517 (w), 1479 (w), 1446 (w), 1388 (w), 1271 (m), 1238 (m), 1196 (m), 1147 (w), 1063 (w), 989 (w), 839 (w), 792 (w), 747 (w).

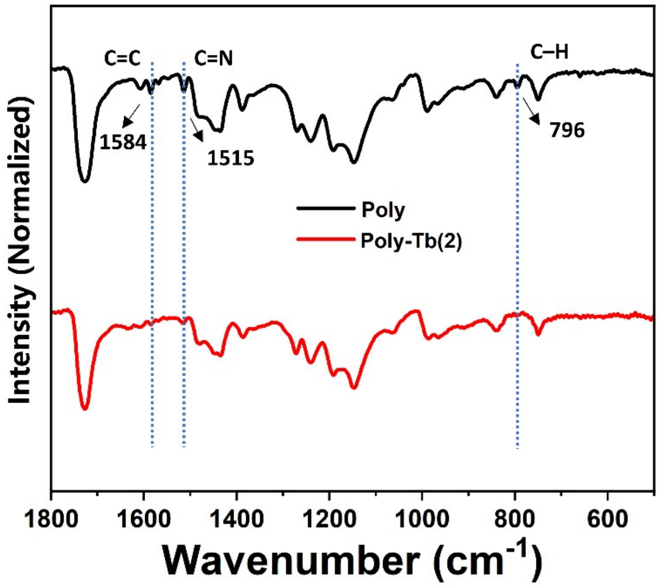

Fig. S6. FT-IR spectrum of Poly and Poly-Tb(2).

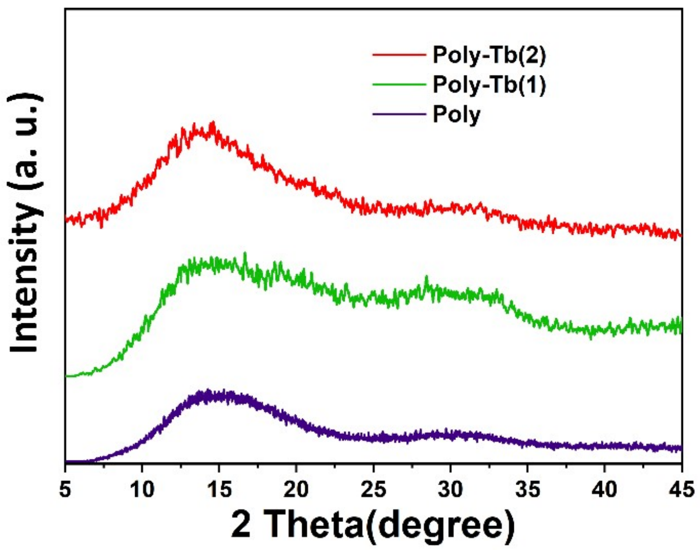

Fig. S7. PXRD patterns of Poly and Poly-Tb(1-2).

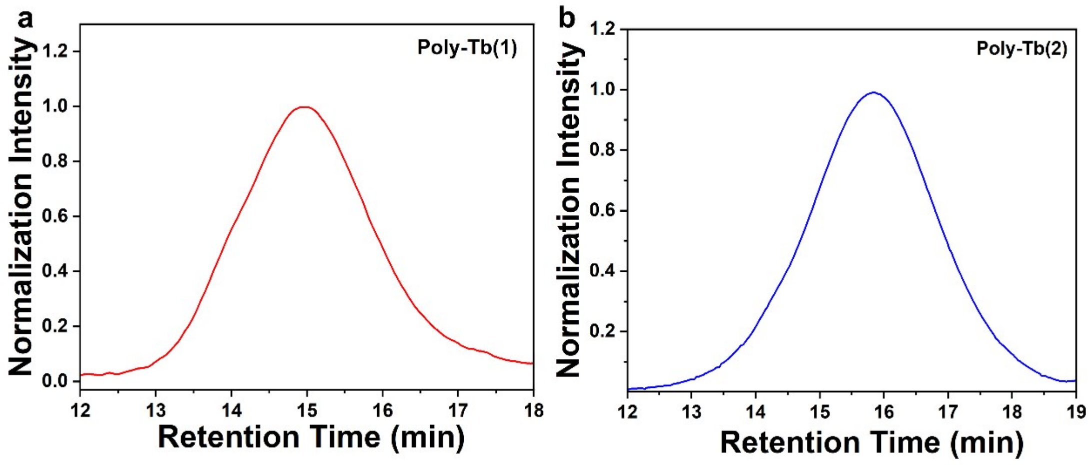

Fig. S8. GPC curves for Poly-Tb(1-2).

Table S1. GPC data for Poly-Tb(1) and Poly-Tb(2).

Polymer	*M*n (kDa)	*M*w (kDa)	*M*z (kDa)	Đ
**Poly-Tb(1)**	12.70	22.80	125.80	1.80
**Poly-Tb(2)**	13.20	28.30	105.80	2.10

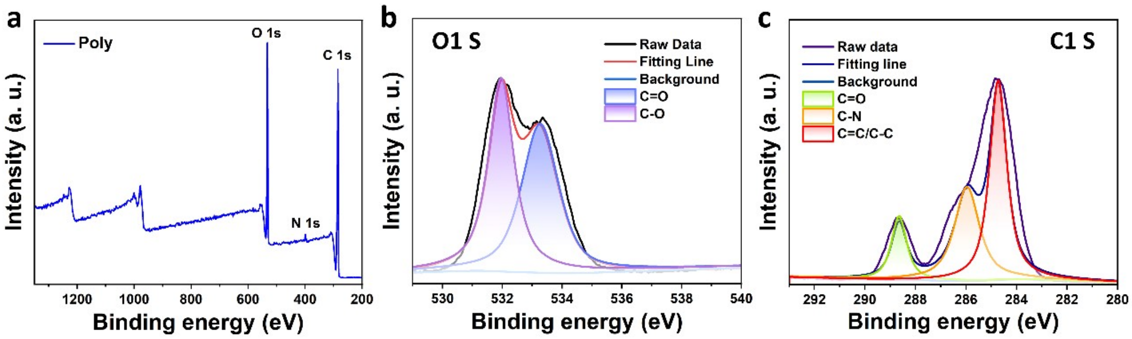

Fig. S9. XPS spectra of Poly.

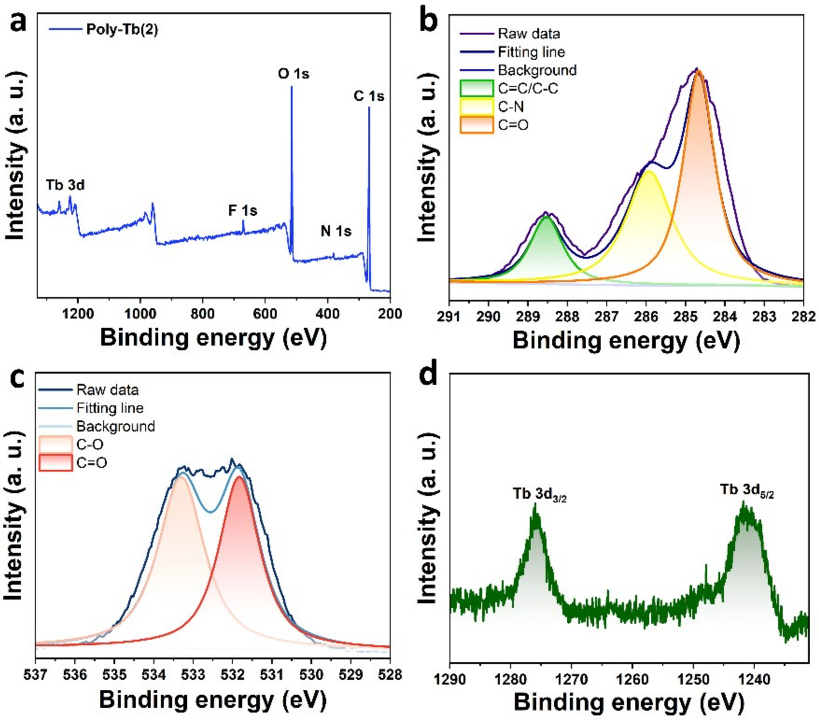

Fig. S10. XPS spectra of Poly-Tb(2).

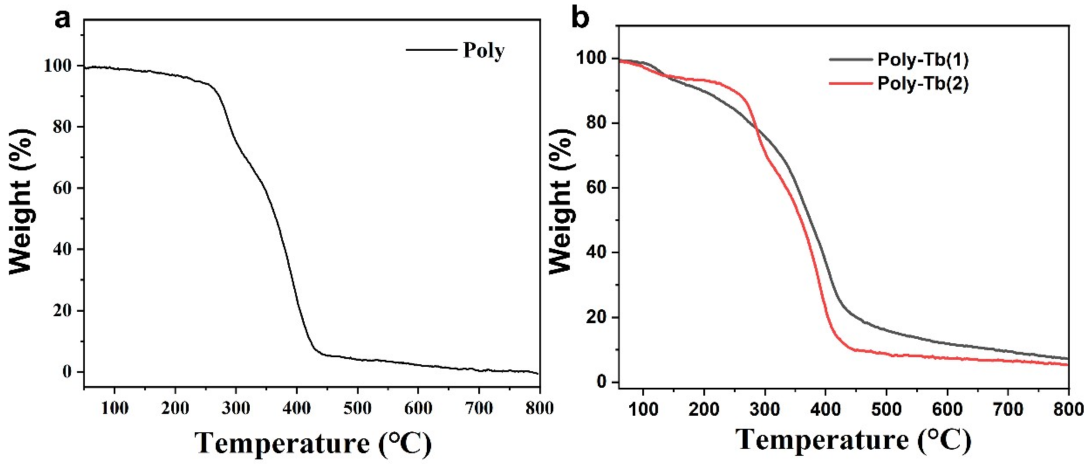

Fig. S11. TGA curves of (a) Poly and (b) Poly-Tb(1) and Poly-Tb(2).

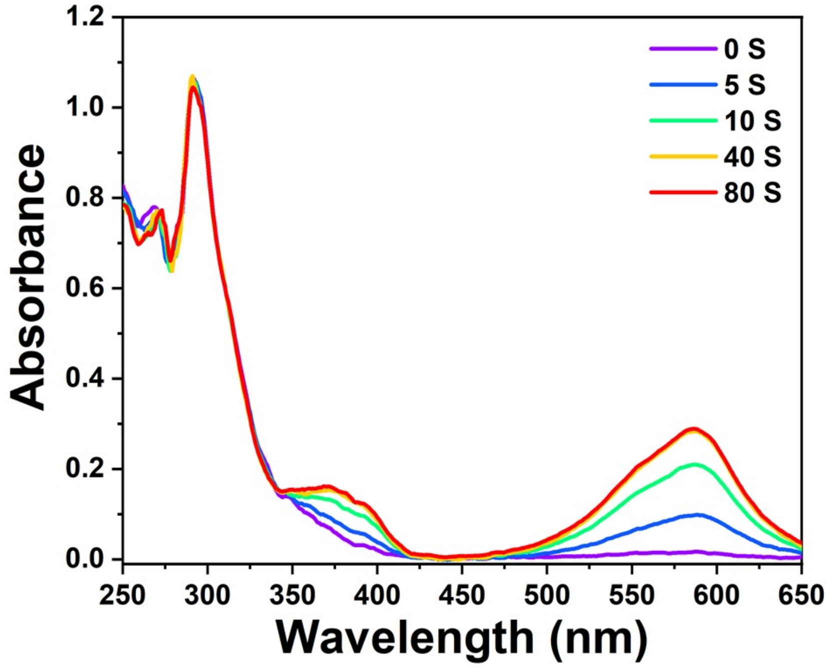

Fig. S12. UV-Vis absorption spectra of Poly-Tb(2) in dichloromethane under different durations of UV irradiation.

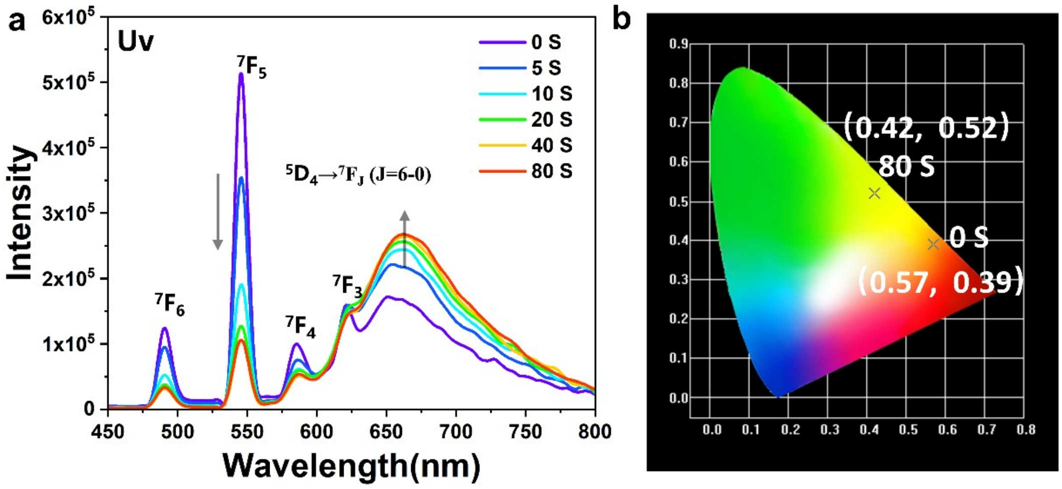

Fig. S13. (a) Photoluminescence (PL) spectra of Poly-Tb(2) recorded after different durations of Uv irradiation and (b) corresponding CIE chromaticity coordinates.

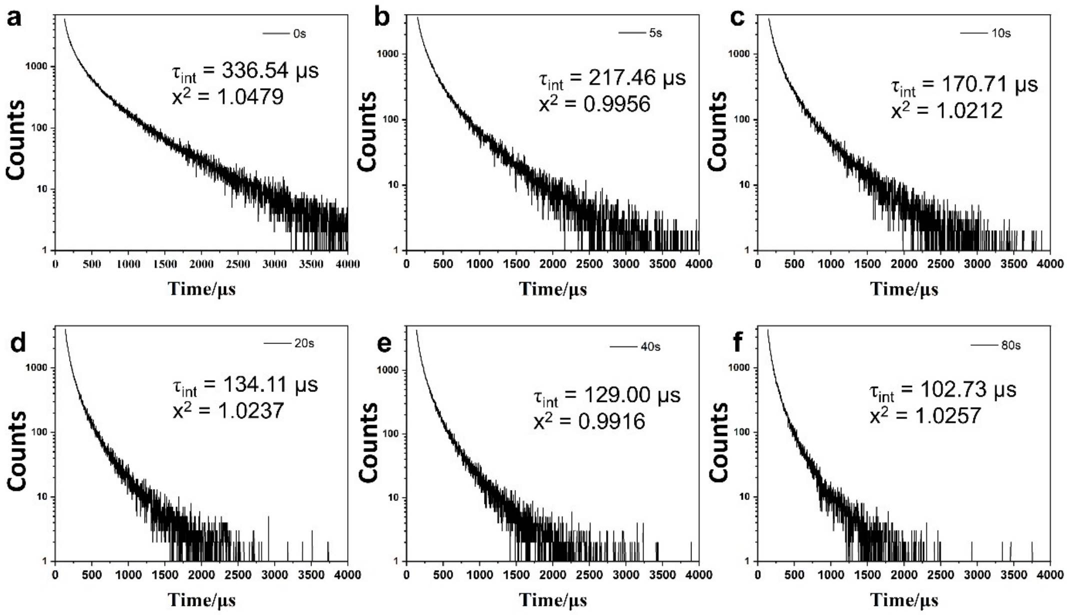

Fig. S14. Luminescence lifetime decay profiles of Tb^{3+} emission from Poly-Tb(1) under different UV irradiation durations (λ_{ex} = 310 nm).

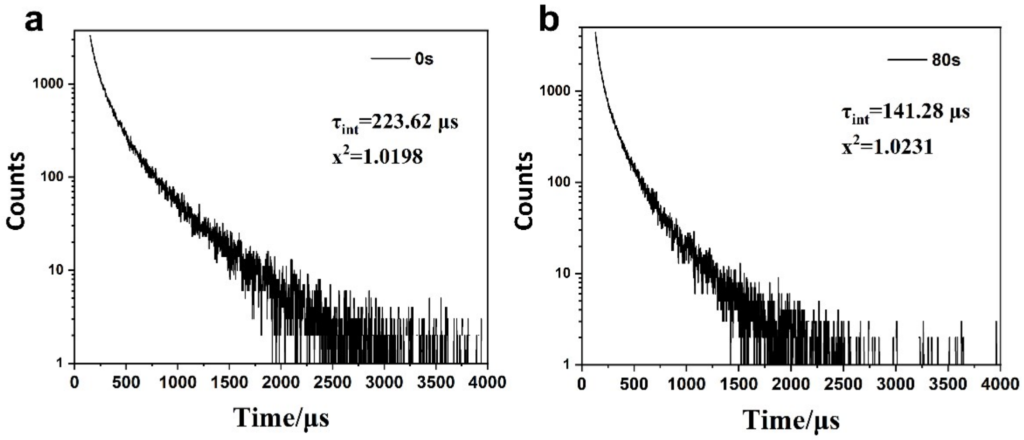

Fig. S15. Luminescence lifetime decay profiles of Tb^{3+} emission from Poly-Tb(2) under different UV irradiation durations (λ_{ex} = 310 nm).

Table S2. Photoluminescence Quantum Yields of Poly-Tb(1) and Poly-Tb(2).

**Polymer**	**λ_{ex} (nm)**	**PLQY (%)**
**Poly-Tb(1)**	310 nm	56.54
**Poly-Tb(2)**	310 nm	45.85

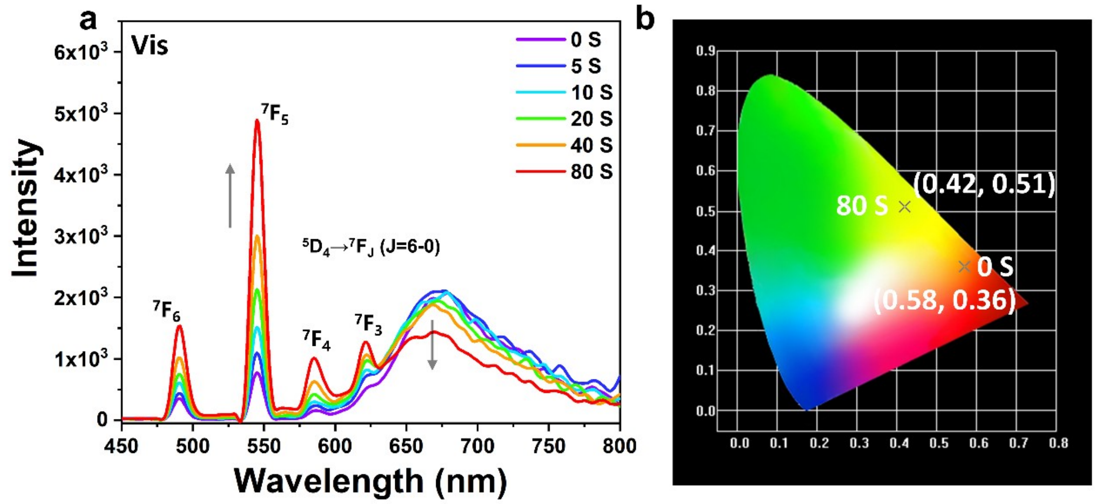

Fig. S16. (a) Photoluminescence (PL) spectra of Poly-Tb(1) recorded after different durations of white light irradiation and (b) corresponding CIE chromaticity coordinates.

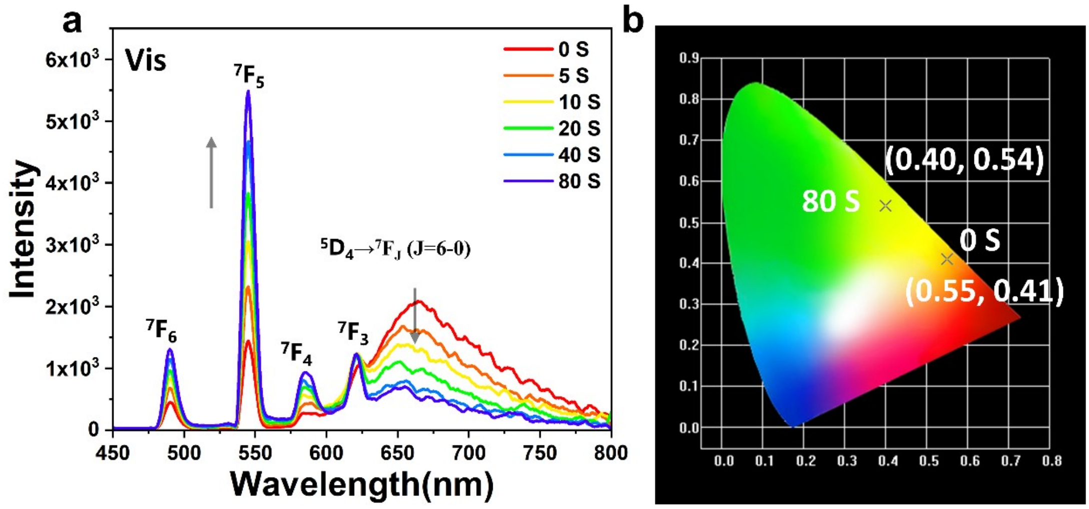

Fig. S17. (a) Photoluminescence (PL) spectra of Poly-Tb(2) recorded after different durations of white light irradiation and (b) corresponding CIE chromaticity coordinates.

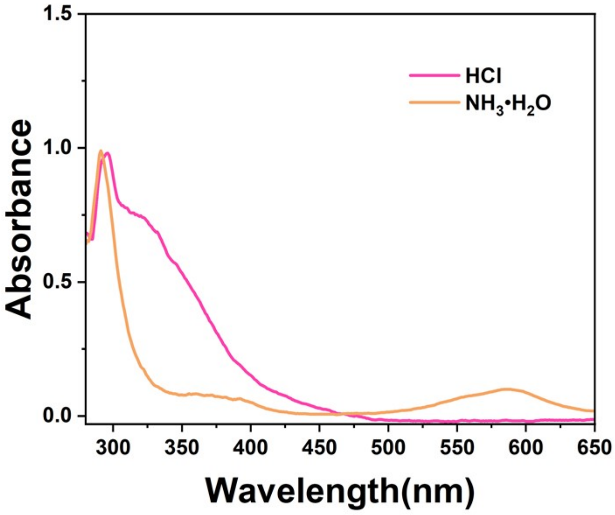

Fig. S18. UV-vis absorption spectra of Poly-Tb(1) (MC state) in solution under acidic and basic conditions.

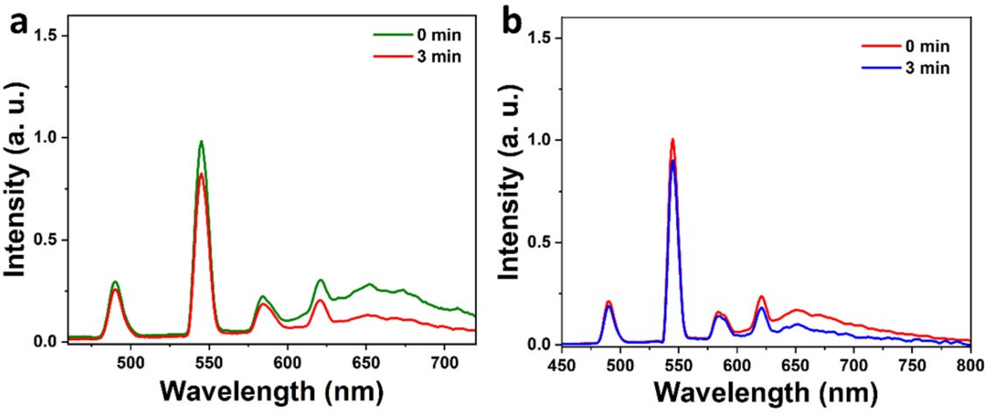

Fig. S19. Emission spectra of (a) Poly-Tb(1) and (b) Poly-Tb(2) in the SP state after fuming with concentration HCl for 3 minutes.

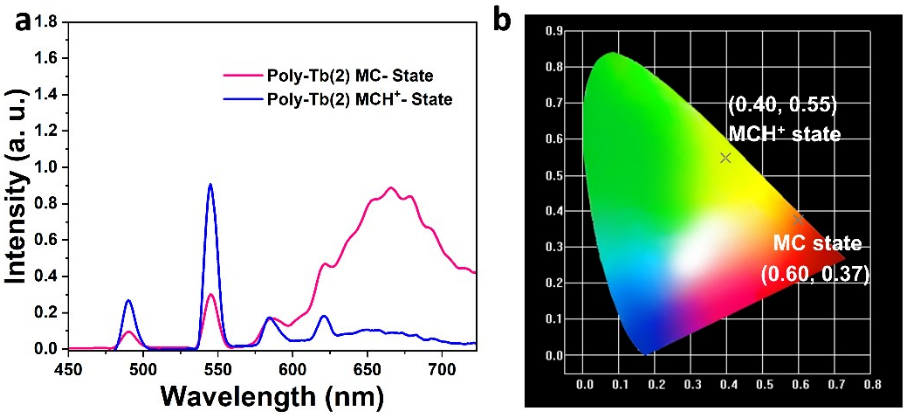

Fig. S20. Emission spectra of Poly-Tb(2) in the MC state after fuming with conc. HCl.

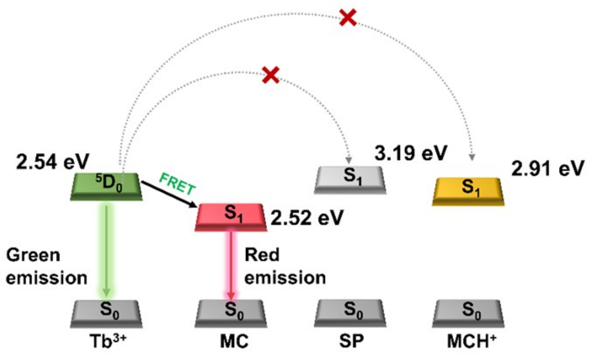

Fig. S21. Simplified Jablonski diagram to explain the Förster energy transfer process between the donor and acceptor in the lanthanide metallopolymer.

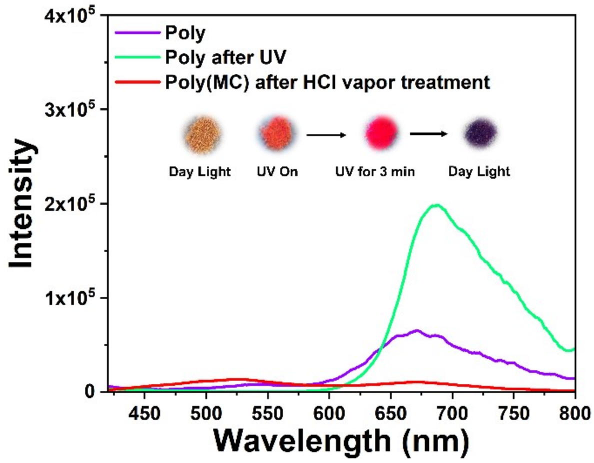

Fig. S22. Emission spectra and fluorescence color changes of the polymer backbone (Poly) under UV light and acid/base stimuli (λ_{em} = 310 nm).

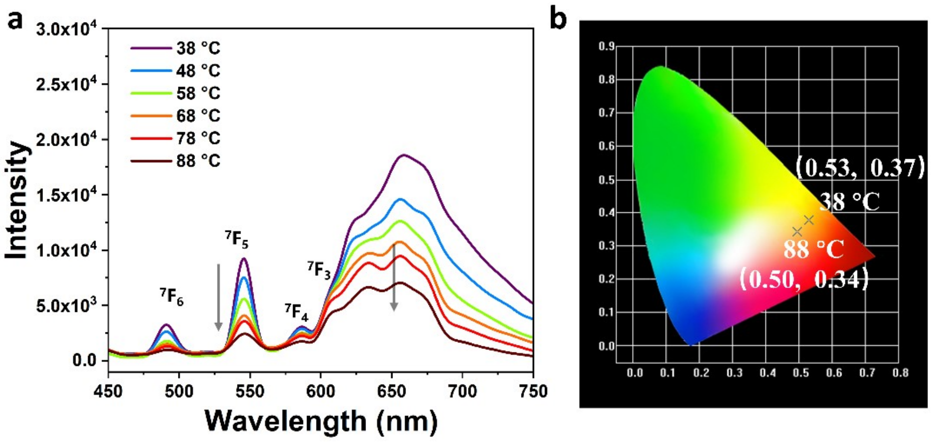

Fig. S23. (a) Photoluminescence (PL) spectra of Poly-Tb(2) (SP state) recorded under different temperature and (b) corresponding CIE chromaticity coordinates.

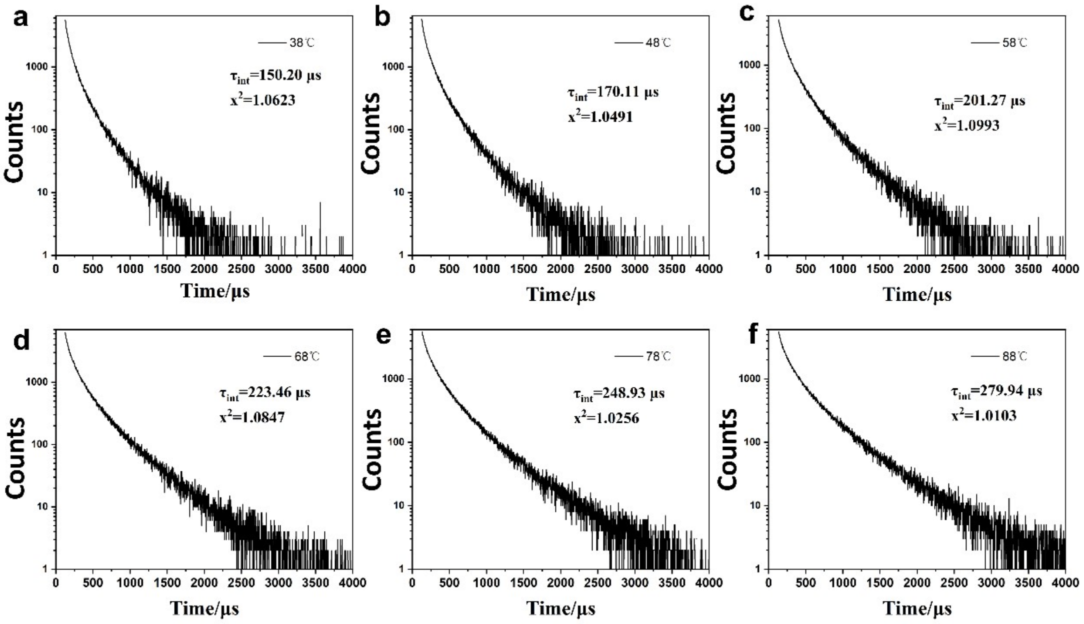

Fig. S24. Luminescence lifetime decay profiles of Tb^{3+} emission from Poly-Tb(1) (MC state) under different temperatures (λ_{ex} = 310 nm).

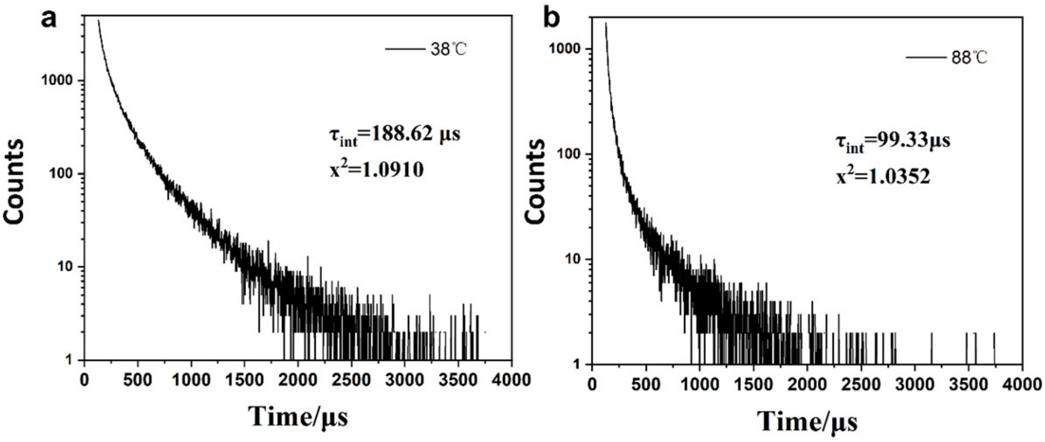

Fig. S25. Luminescence lifetime decay profiles of Tb^{3+} emission from Poly-Tb(2) (MC state) under different temperatures (λ_{ex} = 310 nm).

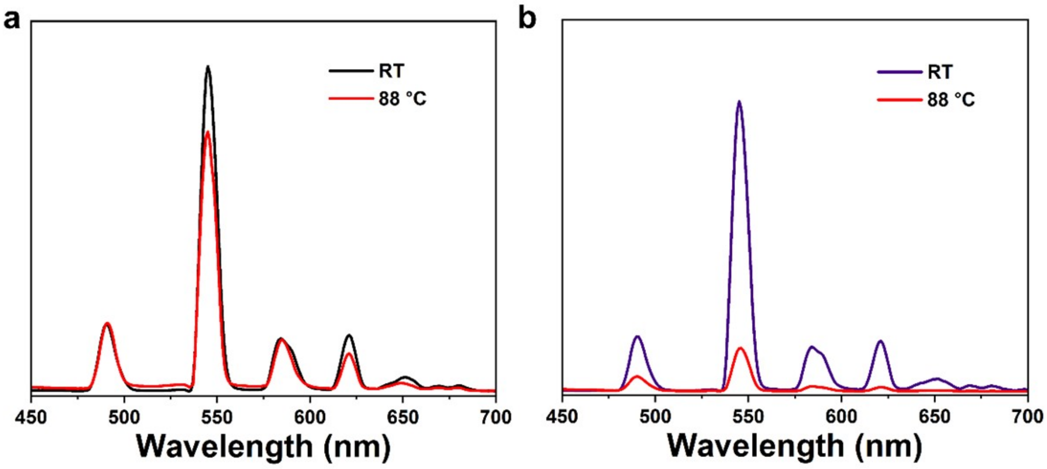

Fig. S26. Photoluminescence (PL) spectra of (a) Poly-Tb(R1) and (b) Poly-Tb(R2) recorded under different temperatures.

References.

S1. Berezin, Y. M; Achilefu, S. *Chem. Rev.* **2010**, *110*, 2641.

S2. Feng, W; Huang, Y; Zhao, Y; Tian, W; Yan, H. *ACS Appl Mater Interfaces,* **2023**, *15*, 17211.

S3. Zhang. H; Li. Q; Yang. Y; Ji. X; Sessler, L. J. *J. Am. Chem. Soc*. **2021**, *143*, 18635.

S4. Liu. J; Song. W; Niu. H; Lu. Y; Yang. H; Li. W; Zhao. Z. Y; Miao. Z. *Inorg. Chem.* **2024**, *63,* 18429.
